# Structure‐based design of peptides that trigger *Streptococcus pneumoniae* cell death

**DOI:** 10.1111/febs.15514

**Published:** 2020-08-31

**Authors:** Sung‐Min Kang, Chenglong Jin, Do‐Hee Kim, Sung Jean Park, Sang‐Woo Han, Bong‐Jin Lee

**Affiliations:** ^1^ Research Institute of Pharmaceutical Sciences, College of Pharmacy Seoul National University Gwanak‐gu Seoul Korea; ^2^ College of Pharmacy Jeju National University Jeju Korea; ^3^ Gachon Institute of Pharmaceutical Sciences, College of Pharmacy Gachon University Incheon Korea; ^4^ Interdisciplinary Graduate Program in Advanced Convergence Technology & Science Jeju National University Jeju Korea

**Keywords:** antibacterial strategy, DNA‐binding, ribonuclease, toxin–antitoxin system

## Abstract

Toxin–antitoxin (TA) systems regulate key cellular functions in bacteria. Here, we report a unique structure of the *Streptococcus pneumoniae* HigBA system and a novel antimicrobial agent that activates HigB toxin, which results in mRNA degradation as an antibacterial strategy. In this study, protein structure‐based peptides were designed and successfully penetrated the *S. pneumoniae* cell membrane and exerted bactericidal activity. This result represents the time during which inhibitors triggered *S. pneumoniae* cell death via the TA system. This discovery is a remarkable milestone in the treatment of antibiotic‐resistant *S. pneumoniae*, and the mechanism of bactericidal activity is completely different from those of current antibiotics. Furthermore, we found that the HigBA complex shows a crossed‐scissor interface with two intermolecular β‐sheets at both the N and C termini of the HigA antitoxin. Our biochemical and structural studies provided valuable information regarding the transcriptional regulation mechanisms associated with the structural variability of HigAs. Our *in vivo* study also revealed the potential catalytic residues of HigB and their functional relationships. An inhibition study with peptides additionally proved that peptide binding may allosterically inhibit HigB activity. Overall, our results provide insights into the molecular basis of HigBA TA systems in *S. pneumoniae*, which can be applied for the development of new antibacterial strategies.

**Databases:**

Structural data are available in the PDB database under the accession number 6AF4.

## Graphical abstract

HigBA complex shows a crossed‐scissor interface with two intermolecular β‐sheets at both the N and C termini of the HigA antitoxin. The crossed‐scissor constitutes a new type of interface between HigB and HigA. We report a unique structure of the *Streptococcus pneumoniae* HigBA system and a novel antimicrobial agent that activates HigB toxin, which results in mRNA degradation as an antibacterial strategy.

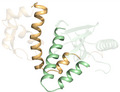

**Abbreviations**
APBS: adaptive poisson‐boltzmann solver
BL: beamline
CD: circular dichroism
CSP: chemical shift perturbation
EMSA: electrophoretic mobility shift assay
HADDOCK: high ambiguity‐driven protein–protein DOCKing algorithm
HSQC: ^1^H‐^15^N heteronuclear single quantum coherence spectroscopy
HTH: helix‐turn‐helix
IPTG: isopropyl‐β‐D‐thiogalactopyranoside
MIC: minimum inhibitory concentration
PDB: protein data bank
PI: propidium iodide
RFU: resulting fluorescence
RMSD: root mean square deviation
SeMet: selenomethionine
TA: toxin–antitoxin
THB: Todd Hewitt Broth
WT: wild‐type

## Introduction

*Streptococcus pneumoniae* is a Gram‐positive species of bacteria that causes disease mainly through respiratory infections [1]. Diseases caused by *pneumococcal* species include otitis, sinusitis meningitis, bacteremia, and pneumonia [2]. The infections caused by *S. pneumoniae* resulted in 515 000 death in 2015 [3], with 335 000 of which in children under 5 years of age [4]. Patients infected with *S. pneumoniae* are mainly treated with antibiotics, such as penicillin, cephalosporins, macrolides, and quinolones [5]. However, the advent of increasing antibiotic resistance strains of *S*. pneumoniae [6] requires the development of novel therapeutics.

Toxin–antitoxin (TA) systems were first discovered as plasmid genes in the 1980s, which is related to the inheritance of plasmids in daughter cells [7]. TA system modules were later found in the most bacterial chromosomes, and their roles in bacterial cells have been intensively studied [8]. TA systems consist of two or more adjacent genes encoding a toxin protein and an antitoxin. Toxins involved in diverse cellular functions, such as protein synthesis inhibition, DNA replication, and cell wall synthesis in response to unfavorable growth conditions. Usually, toxins as ribonucleases degrade mRNA in a specific or nonspecific fashion, and some toxins play roles as gyrase inhibitors and kinases. Their cognate antitoxins neutralize the toxins and regulate the transcription levels of TA operons [9, 10]. Recently, an abundance of TA systems in pathogenic bacteria has been reported by advances in genome sequencing and bioinformatics [11, 12].

TA systems are classified into type I–VI TA systems. Type I TA systems consist of a noncoding RNA antitoxin and a toxin‐encoding mRNA. Type II TA systems consist of a protein antitoxin and its cognate protein toxin. Type III TA systems consist of a small RNA antitoxin that binds directly to the toxin protein as an inhibitor. Similar to the type II TA system, both the toxin and the antitoxin of a type IV TA system are proteins, although their inhibition mechanism is not direct binding. Instead, these proteins play opposing roles in bacterial filaments. In a type V TA system, a ribonuclease antitoxin degrades the mRNA toxin. The protein toxin of a type VI TA system is neutralized by the protein antitoxin, which is degraded by cellular proteases [13].

The most widely distributed type II TA systems comprise the thermodynamically stable toxin and the unstable antitoxin. The antitoxin is cleaved by some cellular proteases and has a lower half‐life than that of the toxin. Therefore, the host depends on a continuous supply of antitoxin to neutralize the toxicities of toxins [14]. Currently, seven type II TA loci have been reported in *S. pneumoniae*: three RelBEs, phd‐doc, HicBA, HigBA, and one unidentified family. Among these seven TA systems, HigBA systems share limited sequence homology among HigB toxins and HigA antitoxins exhibit considerable structural variability [15, 16, 17].

Here, we present the complex structure of HigBA from *S. pneumoniae* as determined by X‐ray crystallography. The crossed‐scissor constitutes a new type of interface between HigB and HigA. The binding interface showed critical residues in the formation of the HigBA complex. HigB from *S. pneumoniae* showed *in vitro* ribonuclease activity, and the mRNA cleavage mechanism was scrutinized through numerous single point mutations. The peptides that mimicked toxin or antitoxin helices could hinder HigBA complex formation. These peptides could penetrate *S. pneumoniae* and exert bactericidal activity. The antitoxin does not block the active site of HigB in the HigBA complex structure, thus revealing that peptide binding may allosterically inhibit HigB activity. HigA has DNA‐binding properties through its helix‐turn‐helix motif (HTH) and regulates HigBA expression. The residues of HigA that are important for DNA binding were investigated by nuclear magnetic resonance (NMR). This approach may contribute to our understanding of the structure–function relationships of TA systems, including the HigBA system. Our biochemical and structural studies will provide valuable information and insights into the molecular basis of TA systems as an antibacterial target.

## Results

### Overall structure of the HigBA complex

**Fig. 1:**
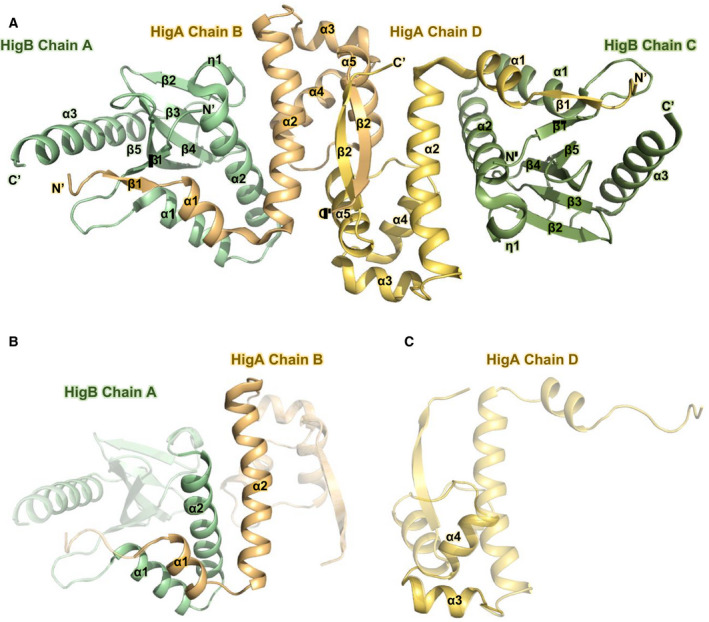
Overall structure of heterotetrameric *S. pneumoniae* HigBA. PyMOL was used to generate Fig. 1. A ribbon representation of the HigBA heterotetramer. Chains A and C of HigB are shown in green and olive, respectively. Chains B and D of HigA are shown in orange and yellow, respectively. The structure reveals a dimeric interface between the C‐terminal β‐strands of the HigA subunits. The β‐strands of HigB (β1‐β5) form antiparallel β sheets. B Binding conformation of the HigBA complex. Interactions between the N‐terminal helices of both HigB and HigA result in a crossed‐scissor conformation at the heterodimeric interface. (C) HTH DNA‐binding motif consisting of α3 and α4 of HigA

The asymmetric unit of the HigBA complex structure is a heterotetramer containing two HigB toxins and two HigA antitoxins (Protein Data Bank (PDB) code 6AF4) (Fig. 1A and Fig. S1A–D). A secondary structural analysis was conducted using the 2Struc server [18]. The oligomeric state of HigBA was determined to be a heterotetramer based on a molecular weight comparison with various reference proteins (Fig. S2).

The *S. pneumoniae* HigB toxin contains three α‐helices, one 3_10_‐helix (η), and five β‐strands in the following order: β1 (residues 4–7), α1 (residues 15–25), α2 (residues 29–48), η1 (residues 49–52), β2 (residues 57–61), β3 (residues 64–67), β4 (residues 72–78), β5 (residues 84–91), and α3 (residues 99–115). The *S. pneumoniae* HigA antitoxin contains five α‐helices and two β‐strands in the following order: β1 (residues 6–9), α1 (residues 10–17), α2 (residues 20–42), α3 (residues 47–54), α4 (residues 58–66), α5 (residues 73–83), and β2 (residues 85–91). The β‐strands of HigB (β1–β5) form antiparallel β‐sheets. The interactions of helices α1 and α2 of both HigB and HigA form a heterodimeric interface (Fig. 1B). Helices α3 and α4 of the HigA antitoxin constitute the HTH DNA‐binding motif [19] (Fig. 1C). The N‐terminal β1‐strands of both HigB and HigA form intermolecular β‐sheets. Because the C‐terminal β‐strands of proximal HigAs bind to form a dimeric interface, two intermolecular β‐sheets form at both the N and C termini of HigA (Fig. 1A).

### Comparative analysis of HigBA structures

**Fig. 2:**
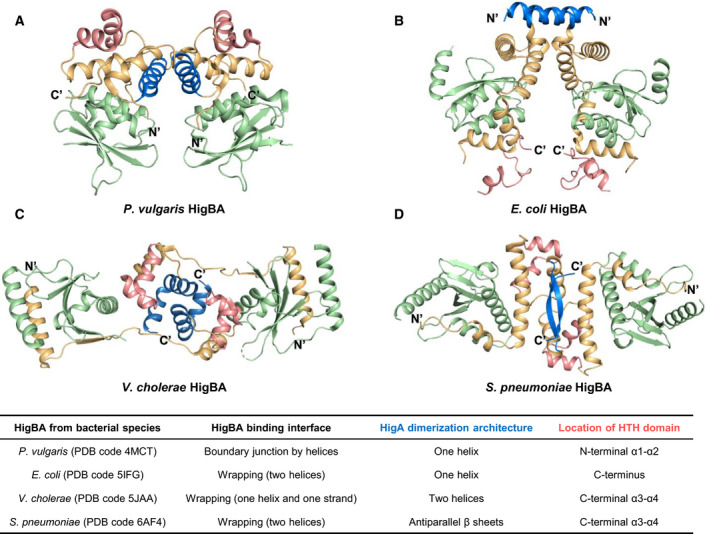
Ribbon representation of the HigBA structures. HigBs and HigAs are shown in green and orange, respectively. HigA dimerization domains are shown in blue, and HigA HTH DNA‐binding domains are shown in red. Structural information is summarized in the table. The N terminus and C terminus of each HigA are also indicated. PyMOL was used to generate Fig. 2. (A) HigBA from *P. vulgaris*. (B) HigBA from *E. coli*. (C) HigBA from *V. cholerae*. (D) HigBA from *S. pneumoniae*

The three previously reported structures of HigBA from *Proteus vulgaris* (PDB code 4MCT) (Fig. 2A) [16], *Escherichia coli* (PDB code 5IFG) (Fig. 2B) [17], and *Vibrio cholera* (PDB code 5JAA) (Fig. 2C) [15] were compared with the newly obtained structure of HigBA from *S. pneumoniae* (PDB code 6AF4) (Fig. 2D). These four structures share the features that each HigB has antiparallel β‐sheets as its main functional unit and each HigA contains an HTH DNA‐binding motif. They form a heterotetrameric assembly, and dimerization is achieved only through the HigAs [HigB‐(HigA)_2_‐HigB]. However, significant differences occur in the complex structures, and the interface between HigB and HigA, the conformation of the HigA dimer, and the position of the DNA‐binding motif of HigA are quite different from each other.

HigAs from *E. coli* and *S. pneumoniae* wrap the cognate HigB with their two helices. HigA from *V. cholerae* wraps its cognate HigB, with one α‐helix and one β‐strand. However, two helices from *P. vulgaris* HigA form boundary junctions with two helices from its cognate HigB. The dimerization domains of HigAs are also quite different from each other. In *P. vulgaris* and *E. coli*, one helix is involved in dimerization, and in *V. cholerae*, two helices are packed to form a dimerization domain. In contrast, in *S. pneumoniae*, an antiparallel β sheet is formed between the C termini of the two subunits. The HTH motif of *P. vulgaris* HigA is located at the N terminus, while the motif of *E. coli* HigBA is at the C terminus. In *V. cholerae* and *S. pneumoniae* HigA, the HTH motif occurs in the middle of the proteins. To understand the differences among HigBAs, a full‐length sequence comparison and the resulting amino acid identity percentages are presented in Fig. S3. Alignments of amino acid residues were carried out using ClustalW [20] and visualized using ESPript 3.0 [21].

### DNA‐binding properties of HigBA

**Fig. 3:**
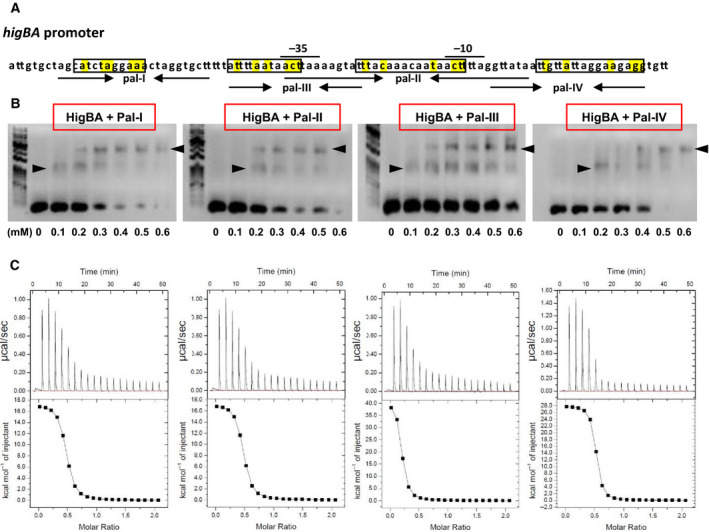
Electrophoretic mobility shift assay and ITC results with palindromes in the *higBA* promoter region. (A) *higBA* promoter region. The putative 10 box and 35 box are denoted. The palindromic sequences Pal‐I, Pal‐II, Pal‐III, and Pal‐IV are shown by arrows facing each other. Consensus recognition sequences are boxed, and identical sequences are highlighted as yellow. (B) EMSAs demonstrated that increasing concentrations of the HigBA complex bound to the four palindromes. Shifted bands are indicated with arrowheads. Each reaction mixtures (10 µL) contained 0.01 mm DNA duplex. Shown data are representative of three independent experiments. (C) ITC assay of four palindromes to which the HigBA complex bound. The binding parameters are described in Table 1

To investigate the DNA‐binding properties of HigBA and HigA, an electrophoretic mobility shift assay (EMSA) experiment was conducted using the four palindromic DNAs of the HigBA upstream region (Pal‐I, Pal‐II, Pal‐III, and Pal‐IV) (Fig. 3A). The palindromic DNAs exhibited a conserved sequence represented as x**a**xx**t**(…)**a**xx**aa** . In the EMSA experiment, the DNA concentration was fixed at 0.01 mm and the protein concentration was increased from 0 to 0.6 mm (HigA dimer and HigBA heterotetramer). As the amount of added protein increased, the bands of the DNA–protein complex shifted upward, showing that HigBA binds to all four DNAs (Pal‐I, Pal‐II, Pal‐III, and Pal‐IV) (Fig. 3B). In the EMSA results, two complexes showing distinct mobility were observed. We surmised that HigBA binds to DNA, which results in the formation of a lower‐shifted band. However, because the DNA sequences are relatively long (at least 20mer), two HigBA heterotetramers might be able to bind to one DNA. Therefore, an increase in the fraction of the super‐shifted band likely occurred at higher concentrations of HigBA. HigBA showed the highest affinity for Pal‐III among the four DNAs. To monitor the binding specificity of HigBA to its upstream promoter, an EMSA experiment was also conducted using the other two palindromic sequences ‘A’ and ‘B’, which did not belong to the promoter region of HigBA. The results showed that band shifts did not occur in the cases of ‘A’ and ‘B’ or the control DNA ‘X’, indicating a lack of DNA–protein complex formation between the proteins and DNA (Supplementary Fig. S4). An isothermal titration calorimetry (ITC) experiment was conducted to estimate the binding affinity of HigBA (Fig. 3C). The DNA‐binding reaction of HigBA is endothermic and entropically driven, and the thermodynamic parameters are listed in Table 1. The binding stoichiometry (*n*) indicates that one HigBA heterotetramer binds to each DNA duplex. The thermodynamic parameters of HigA are provided in Fig. S5. The experimental results showed that the HigBA complex has a higher affinity for DNA than the HigA complex based on the *K_d_* values. Other ITC experiments were also performed in the absence of proteins under the same experimental conditions. No calorimetric changes were observed in the control experiments using a protein‐free buffer. In addition, no thermal changes were observed in the experimental injections of control DNA ‘X’ or those with the palindromic sequences ‘A’ and ‘B’.

**Table 1:** Thermodynamic parameters upon the binding of DNA to HigBA

DNA	*n (*the binding stoichiometry*)*	*K*_d_ (μm)	*△H* (kcal mol^−1^)	*T△S* (kcal mol^−1^)	*△G* (kcal mol^−1^)
Pal‐I	0.53 ± 0.07	0.46 ± 0.03	2.8 ± 0.4	8.74	−5.94
Pal‐II	0.49 ± 0.06	0.70 ± 0.08	3.6 ± 0.3	7.37	−3.77
Pal‐III	0.48 ± 0.04	0.20 ± 0.01	1.7 ± 0.2	8.14	−6.44
Pal‐IV	0.48 ± 0.05	1.19 ± 0.14	4.3 ± 0.3	9.73	−5.43

**Fig. 4:**
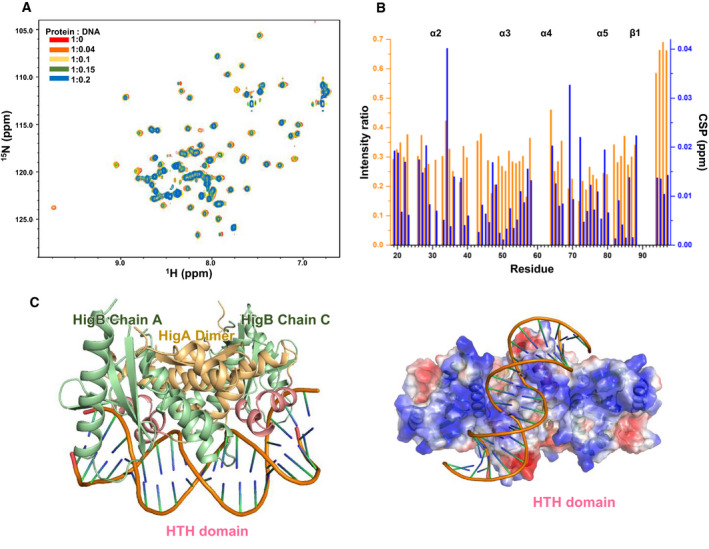
DNA‐binding properties determined from NMR titration data and *in silico* docking model of the HigBA‐DNA complex. (A) NMR titration of HigA^19‐97^ with Pal‐III DNA. 2D‐^1^H, ^15^N HSQC spectra of HigA^19‐97^ (0.5 mm) acquired at varying Pal‐III DNA concentrations (0 to 0.1 mm) are overlaid. The ratios and color coding are shown in the figure. (B) Intensity ratio (orange columns) and CSP value (blue columns) plotted against the residue number. (C) *In silico* docking model of the HigBA‐DNA complex (left) with the electrostatic potential surface (right). HTH DNA‐binding domains of HigA are shown in red. PyMOL was used to generate Fig. 4C

To determine the DNA‐binding site of HigA, a NMR titration experiment on HigA was conducted with Pal‐III DNA, which had the highest affinity with HigA. Because of the poor quality of the spectrum of full‐length HigA (Fig. S6A), HigA^19–97^ was used for this experiment. The 63 N‐H peaks in the ^1^H‐^15^N heteronuclear single quantum coherence spectroscopy (HSQC) spectra were assigned to individual residues of HigA^19–97^ (Fig. S6B). The oligomeric state of the deletion mutant HigA^19–97^ was determined to be a homodimer and showed an unchanged binding mode (Fig. S2). Then, the HSQC spectra of HigA^19–97^ (0.5 mm) with increasing concentrations of Pal‐III (0 to 0.1 mm) were overlaid (Fig. 4A). The ^1^H‐^15^N HSQC peaks of HigA showed no significant chemical shift changes with decreased intensity as illustrated in Fig. 4B. The intensity reduction and disappearance of the ^1^H‐^15^N HSQC cross‐peak of HigA may originate from the increased transverse relaxation rate of HigA^19–27^ by complex formation with Pal‐III DNA. Although small chemical shift changes were observed, S34, T69, Q72, and A88 showed relatively distinct chemical shift changes compared with the other residues (Fig. 4B) as well as large intensity reductions. Although the overall cross‐peaks exhibited intensity reduction, the largest effects for ^1^H‐^15^N HSQC cross‐peak intensity reduction were found in helices α3, α4, and α5 and their connecting loops. Helices α3 and α4 form the HTH motif, which is strongly related to DNA binding. In addition, α5 and its connecting loop are close to the HTH motif.

Based on these results, the binding pattern between DNA and HigBA was deduced by HADDOCK. Multiple solutions of the in silico docking model of the HigBA‐DNA complex were clustered automatically to obtain the best single cluster of docking results. The best solution showed a score of −207.9 ± 2.7, the smallest root mean square deviation (RMSD) (2.2 Å ± 0.3 Å), and the largest buried surface area (2458.2 ± 38.0 Å^2^) (Fig. 4C). Helices α3 and α4 of HigA constituting the HTH motif bind mainly to the major groove of the DNA. In the electrostatically generated interface, α3 exhibited a negative charge while α4 exhibited a positive charge.

### Active site of HigB deduced from homologs

**Fig. 5:**
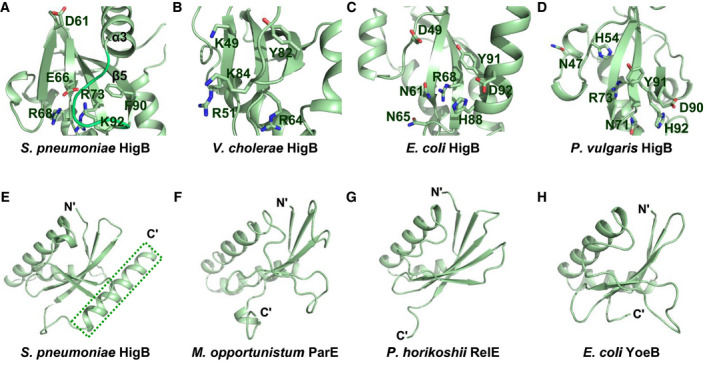
Active site residues of *S. pneumoniae* HigB compared to those of other HigBs, and overall structures of ribonucleases from similar families. PyMOL was used to generate Fig. 5. (A–D) Active site residues of *S. pneumoniae* HigB compared to those of other HigBs. The HigB active site residues of (A) *S. pneumoniae*, (B) *V. cholerae*, (C) *E. coli,* and (D) *P. vulgaris*. The active sites of HigBs are oriented to show the antiparallel β‐sheets in frontal view. (E–H) Overall structures of ribonucleases from similar families. The C‐terminal α‐helix observed only in *S. pneumoniae* HigB is highlighted in a green dashed box. (E) *S. pneumoniae* HigB, (F) *M. opportunistum* ParE, (G) *P. horikoshii* RelE, and (H) *E. coli* YoeB

HigB toxins contain several conserved active site residues that are essential for the cleavage of mRNA. For a detailed comparison of *S. pneumoniae* HigB (Fig. 5A and E) with other HigBs, the structural similarities of HigBs were analyzed using Dali [22], and the reported active site residues were arranged. Despite low statistical similarity, the active sites of HigBs were found to be located in their antiparallel β‐sheets. The detailed Dali search results with HigB from *Vibrio cholera* [15] (Fig. 5B), HigB from *E. coli* [17] (Fig. 5C), and HigB from *Proteus vulgaris* [16] (Fig. 5D) are shown in Table 2. According to the structural alignment, the putative active residues of *S. pneumoniae* HigB for ribonuclease activity may be D61, E66, R68, R73, F90, and K92. Compared with other HigBs, the active site residues of *S. pneumoniae* HigB are sterically hindered by a flexible loop between β5 and α3.

**Table 2:** Structural similarity comparison of HigB with its homologs using the Dali server

Protein name	Source	PDB code (used chain)	Z‐score	RMSD (Å)	Number of aligned Cα	Sequence identity (%)
HigB	*V*. *cholera*	5JAA (D)	6.8	3.6	87	10
HigB	*E*. *coli*	5IFG (A)	6.9	2.9	83	4
HigB	*P*. *vulgaris*	4MCT (D)	6.4	2.7	77	14

In addition, the overall structures of ribonucleases from similar families were compared using Dali, which showed high statistical values for ParE from *Mesorhizobium opportunistum* [23] (Fig. 5F), RelE from *Pyrococcus horikoshii* [24] (Fig. 5G), and YoeB from *E. coli* [25] (Fig. 5H) (Table 3). These ribonucleases showed significant structural similarity despite their low sequence identity. The C‐terminal α‐helix in *S. pneumoniae* HigB is not conserved in other structural homologs.

**Table 3:** Structural similarity comparison of HigB with its homologs using the Dali server

Protein name	Source	PDB code (used chain)	Z‐score	RMSD (Å)	Number of aligned Cα	Sequence identity (%)
ParE	*M*. *opportunistum*	5CEG (D)	9.8	2.4	86	3
RelE	*P*. *horikoshii*	1WMI (A)	9.8	1.8	80	11
YoeB	*E*. *coli*	2A6R (D)	9.8	2.0	79	16

### Intermolecular interaction of HigBA

**Fig. 6:**
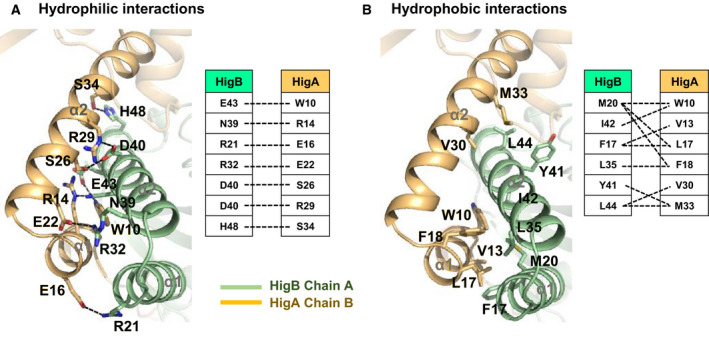
Crossed‐scissor heterodimeric interface between HigB and HigA. The same colors as in Fig. 1 are employed. PyMOL was used to generate Fig. 6. (A) Residues involved in hydrophilic interactions at the interface. Hydrogen bonds and salt bridges are shown as black dotted lines. The residues participating in hydrophilic interactions are also shown in stick representation. (B) Residues involved in hydrophobic interactions at the interface. The residues participating in hydrophobic interactions are also shown in stick models. (A,B) Schematic diagrams of the hydrophilic interactions (left) and hydrophobic interactions (right) contributed by residues in the heterodimeric interface of HigBA

Substantial intermolecular networks between HigB and HigA are formed at the cross‐scissor interface. Helices α1 and α2 of both HigB and HigA underwent hydrophilic and hydrophobic interactions to form binding interfaces. In the interface, the residues of HigB involved in hydrophilic interactions are R21, R32, N39, D40, E43, and H48, and they interact with W10, R14, E16, E22, S26, R29, and S34 of HigA. D40 of HigB has the closest interactions with S26 and R29 of HigA (Fig. 6A). Hydrophobic interactions are contributed by F17, M20, L35, Y41, I42, and L44 of HigB and by W10, V13, L17, F18, V30, and M33 of HigA. F17, M20, and L44 of HigB have hydrophobic interactions with multiple counterparts (Fig. 6B). These binding networks are summarized in Fig. 6A and B.

### Ribonuclease activities of HigB and its mutants

**Fig. 7:**
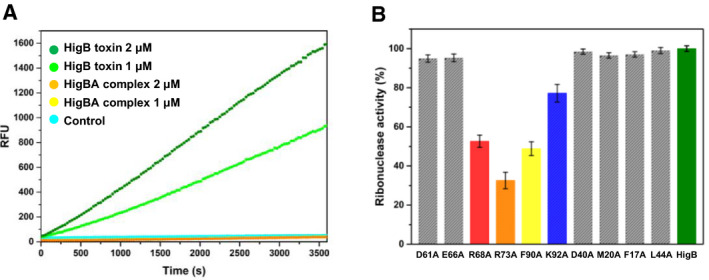
*In vitro* ribonuclease activity assays of *S. pneumoniae* HigB with its mutants. Each experiment was performed in triplicate. (A,B) *In vitro* ribonuclease activity assays of *S. pneumoniae* HigB and comparison of the ribonuclease activity with that of HigB mutants. (A) Fluorescence measurements as a function of time. The control did not show activity, and HigB showed concentration‐dependent ribonuclease activity. (B) Comparison of the ribonuclease activity of the WT HigB with those of the HigB mutants. A WT HigB at 2 μm was used as a reference for comparison. The concentration of each HigB mutant was the same as that of the WT HigB. The RFU obtained with the reference was taken as 100%. Arg73 of HigB showed the strongest effect on the ribonuclease activity of HigB. Error bars represent the standard deviations of the three replicate reactions

We performed ribonuclease activity assays on HigB using fluorescence‐labeled RNA substrates. According to amount of the substrate cleaved by HigB, fluorescence is proportionally emitted. The ribonuclease activity of *S. pneumoniae* HigB is verified through the increase in resulting fluorescence (RFU) as a function of time and concentration (Fig. 7A). The control containing only RNA substrate without proteins showed no ribonuclease activity.

In addition, to determining the mutation effects of active site residues and the main residues interacting with HigA, ribonuclease activity was also measured for a total of 10 single amino acid mutants (mutations in active site residues D61A, E66A, R68A, R73A, F90A, and K92A and HigA‐interacting residues D40A, M20A, F17A, and L44A) (Fig. 7B). R73A showed approximately 70% less ribonuclease activity than the wild‐type (WT) HigB, and F90A and R68A also showed significant reductions in ribonuclease activity. The mutation effect of K92 (K92A) was relatively reduced, and the mutations of D61, E66, and the HigA‐interacting residues had almost negligible effects on ribonuclease activity. The secondary structural integrity of HigB and the HigB mutants was confirmed by circular dichroism (CD) spectroscopy (Supplementary Fig. S7A).

**Fig. 8:**
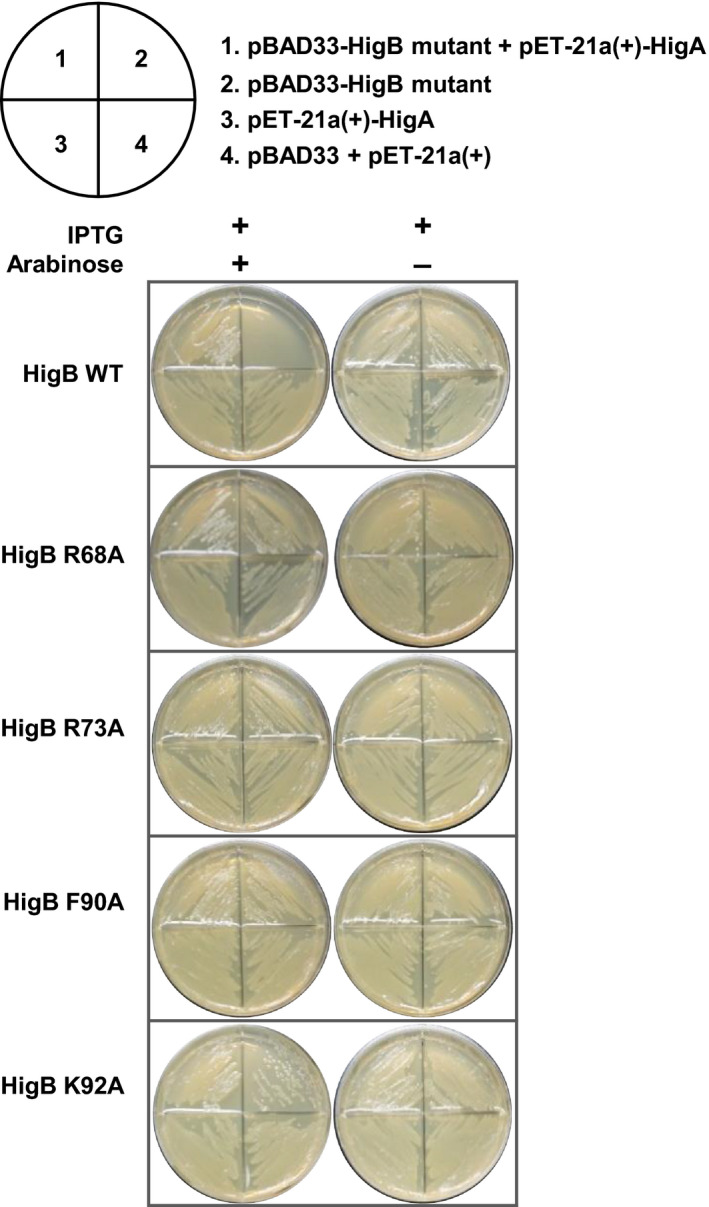
Toxicity and antitoxicity assay of HigB variants and their combinations with HigA in *E. coli*. Each quadrant contains the corresponding plasmids indicated above. The plate on the left contained both L‐arabinose and IPTG that induced HigB (toxin) variants and HigA (antitoxin) expression, whereas the plate on the right contained IPTG that induced only antitoxin expression. The expression of plasmids encoding the WT HigB (using the pBAD33 vector) resulted in cell growth arrest; the toxicity was neutralized by coexpression of the plasmid encoding HigA (using the pET‐21(a) vector). The mutations of R69, R73, and F90 reduced the activity of the HigB toxin, and the mutation of K92 slightly reduced the activity of HigB toxin (segment 2). The HigA‐induced toxicity inhibition can be confirmed via good growth

Based on these *in vitro* results, four efficient mutants (R68A, R73A, F90A, and K92A) showing significant reductions in ribonuclease activity were validated through an *in vivo* toxicity neutralization test. To perform this kill‐and‐rescue assay using *E. coli*, HigB and its mutants were cloned into the L‐arabinose‐inducible vector pBAD33 and HigA was cloned into the IPTG inducible vector pET‐21a(+). As expected from the *in vitro* test results, the toxicity of HigB was efficiently rescued by HigA and HigB mutations of residues R68, R73, and F90. The K92A mutant exhibited slightly weak toxicity compared to the WT HigB, showing that the toxicity of HigB was not strongly affected by the mutation of K92 compared to mutations of the other residues (R68, R73, and F90) (Fig. 8). This assay demonstrated that HigB is a *bona fide* toxin with cytotoxicity and that the active site of HigB has been successfully deduced.

### HigBA complex disruption by mimicking peptide

Peptides mimicking the binding region between HigB and HigA were added to disrupt the HigBA complex. When the HigBA complex is disrupted by peptides and free HigB is released, fluorescence‐labeled RNA substrates emit fluorescence in proportion to the amount of the substrate cleaved by HigB. The α‐helical nature of one HigB‐mimicking peptide and two HigA‐mimicking peptides was confirmed by CD spectroscopy (Fig. S7B).

**Fig. 9:**
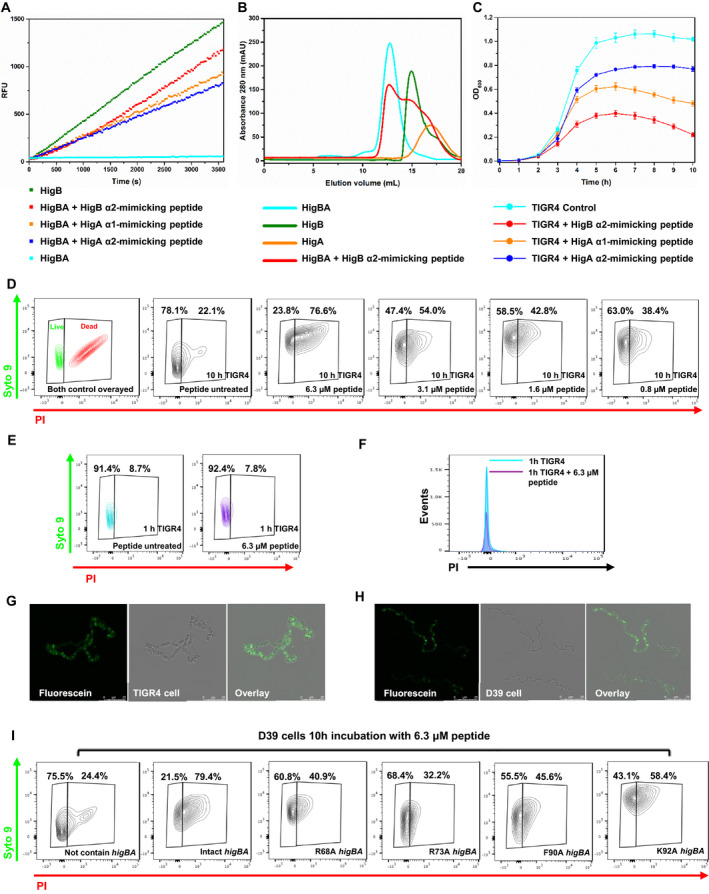
Protein complex disruption and subsequent *in vivo* cell feasibility assays using binding region‐mimicking peptides. (A) *In vitro* ribonuclease activity assays using binding region‐mimicking peptides. Each experiment was performed in triplicate. Each mimicking peptide (10 μm) was added to 2 μm HigBA. Forty units of RiboLock^TM^ (Thermo Scientific) RNase inhibitor was used to prevent ribonuclease contamination. (B) Size‐exclusion chromatography of HigBA, HigA, HigB, and HigB with the HigB α2 helix‐mimicking peptide added. Absorption at 280 nm is plotted as a function of the elution volume. The volume of injected protein was 100 μL, and the concentrations of proteins and HigB α2‐mimicking peptide were 200 μm and 1 mm, respectively. (C) Effect of peptides on the growth of *S. pneumoniae* TIGR4 was measured based on the OD_600_. Error bars represent the standard error of the mean across three biological replicates. (D–I) Cell viability confirmation using flow cytometry and confocal imaging. (D) *S. pneumoniae* TIGR4 was treated with various concentrations of HigB α2 mimicking peptide selected by the results of the MIC test. Cells were labeled with LIVE/DEAD BacLight stains (Syto 9; PI) and analyzed by fluorescence‐activated cell sorting (FACS). The results of the untreated peptide cells are also presented for comparison. Live cell/dead cell areas were established using this TIGR4 control. (E) FACS data of 6.3 μm peptide‐treated *S. pneumoniae* TIGR4 after 1 h of incubation. (F) Flow cytometry of *S. pneumoniae* TIGR4 cells obtained from untreated (cyan) and 6.3 μm peptide‐treated (purple) cells stained with the fluorescent dye PI. Detection of cell‐penetrating peptides in living cells by confocal laser scanning microscopy using (G) *S. pneumoniae* TIGR4 and (H) *S. pneumoniae* D39 containing active site‐mutated *higBA* (shown data are images of R73A). (G,H) Shown data are representative maximum intensity Z projection images of three independent experiments with scale bar, 25 μm. (I) FACS data of *S. pneumoniae* D39 that was treated with 6.3 μm peptide and contained the active site‐mutated *higBA*

The addition of mimicking peptides resulted in the effective disruption of HigBA, showing regeneration of the toxicity of peptide‐treated HigBA compared to the activity of HigB (Fig. 9A). When the HigB α2 helix‐mimicking peptide was added to HigBA, the ribonuclease activity reached approximately 80% of that of HigB. Subsequently, the complex disruption of the HigB‐mimicking peptide was also verified by chromatograms using a Superdex 75 10/300 prepacked column (GE Healthcare, Chicago, IL, USA) (Fig. 9B).

### Artificial activation of toxin by designed peptides can exert bactericidal effects *in vivo*

To support the above results in an *in vitro* enzyme‐level experiment, diverse *in vivo* studies were performed. An antimicrobial activity test of the mimicking peptides against *S. pneumoniae* TIGR4 revealed that cell growth was inhibited at concentration of 6.3 μm HigA α1‐, HigA α2‐, or HigB α2‐mimicking peptide. A cell growth assay of the designed peptides at 6.3 μm showed the highest growth defects in the presence of the HigB α2‐mimicking peptide (Fig. 9C). Scrambled peptides with the same amino acid sequence as the HigB α2‐mimicking peptide did not inhibit *S. pneumoniae* cell growth.

We then tested the effect of HigB α2‐mimicking peptide addition on *S. pneumoniae* TIGR4 cells by the flow cytometry analysis of cells labeled with LIVE/DEAD BacLight stains (Fig. 9D). In the absence of peptides, 22.1% of *S. pneumoniae* TIGR4 cells were permeable to propidium iodide (PI). In contrast, for *S. pneumoniae* TIGR4 cells treated with peptides, the proportion of PI‐permeable cells increased to 78.1% in the presence of 6.3 μm peptides after 10 h of incubation, indicating the bactericidal activity of the structure‐based designed peptides. To support the suggested mode of killing, the data after 1 h of incubation with peptides are provided (Fig. 9E and F). The results show that most cells lived after 1 h of incubation. Thus, the peptides might not have caused the loss of membrane potential and membrane barrier function.

The successful intracellular localization of peptides was also demonstrated by confocal microscopy using fluorescein‐labeled peptides (Fig. 9G). Fluorescein‐only (left) and bright‐field images of *S. pneumoniae* TIGR4 (middle) are presented. An overlaid image (right) indicates the successful intracellular localization of the peptide. Because surface‐bound peptides and extracellular peptides were degraded by trypsin, the images indicate that the fluorescence signals are indeed mediated by peptide internalization. Because confocal images were taken after 1 h of incubation with the peptides, the peptides were introduced much earlier than PI and peptide passage might have allowed the cytoplasmic membrane to maintain its barrier and energy‐generating function for quite some time after introducing the peptide.

For further proof of the suggested killing mechanism, *S. pneumoniae* D39 without *higBA* and *S. pneumoniae* D39 containing intact *higBA* or active site‐mutated *higBA* were treated with 6.3 μm peptides. After 1 h, the peptides entered the *S. pneumoniae* D39 cells containing active site‐mutated *higBA*, and the results were consistent with that of *S. pneumoniae* TIGR4 (Fig. 9H). However, after 10 h, *S. pneumoniae* D39s containing active site‐mutated *higBA* presented longer lifespans than *S. pneumoniae* D39s containing intact *higBA* (Fig. 9I). *S. pneumoniae* D39s that did not contain *higBA* seemed to be unaffected by peptides.

## Discussion

### Insights into the transcription regulation mechanism of the HigBA system

Although many other proteins present HTH DNA‐binding motifs, antitoxins with HTH motifs in the HigBA system not only reveal differences in the location of their DNA‐binding motifs but also show functional differences. HigA antitoxins have almost no structural similarity to other proteins with an HTH motif, such as the domain position, dimerization mechanism, and binding pattern to the cognate HigB toxin. The structural variability of these HigA antitoxins will cause differences in their transcriptional regulation. In the case of *S. pneumoniae* HigA, the binding between DNA and HigA is dominated by certain residues belonging to the HTH motif and their neighboring residues. When DNA was added to HigA through titration, these residues showed an overall reduction in NMR peak intensity, which shows that these residues are affected by DNA binding.

HigBA and HigA from *S. pneumoniae* seem to have different binding affinities for the four palindromic sequences tested. HigA has been suggested to regulate transcription by sequence‐specific interactions with DNA and by multisite binding [26]. In addition, our experimental data show that the HigBA complex has higher binding affinity to DNA than to HigA antitoxin alone. Because the affinities of HigA to Pal‐I, Pal‐II, and Pal‐III are weak compared with those of HigBA, the DNA‐binding modes of HigA and the HigBA complex will differ. Additionally, an operator appears to be preferentially recognized when the HigBA complex is formed. Therefore, there might be more than one transcriptional regulation mechanism. In general, the diversity of transcriptional regulation mechanisms due to the structural variability of HigA antitoxins is likely a key aspect of the transcription regulation mechanism governed by the HigBA TA system.

### Application of structural studies to the catalytic mechanism of HigB

HigB from *S. pneumoniae* is structurally homologous to the ribosome‐dependent toxin RelE [27]; however, experimental results showed possible similarities to the ribosome‐independent toxin YoeB [25]. Since HigBA system proteins share very low sequence similarity, many proteins belonging to the HigBA system have been elucidated via structural studies [15, 16, 17]. Therefore, the deposition of structural information regarding additional HigBA proteins in the database may allow for confirmation of whether the C‐terminal helix present in HigB is related to target specificity. Knowledge about the regulation of type II toxin‐mediated cleavage could explain the mechanism underlying the cellular responses of bacteria to environmental changes [27, 28]. Furthermore, recent studies demonstrated that type II toxins play crucial roles in bacteria and participate in various functions, such as drug resistance and persistent cell formation [29, 30]. Therefore, understanding the molecular mechanism of RNA recognition can provide fundamental principles for understanding toxin function. Residues that have a major role in the activity of HigB have been proposed according to the results of structural and functional studies; however, data on other types of HigB are needed to understand the detailed mechanisms, such as substrate recognition and catalytic stabilization.

The active site of HigB from *S. pneumoniae* is positively charged, suggesting possible interactions with the negatively charged RNA phosphate backbone (Fig. S8). As evidenced by the mutational study, the following four key residues participate in catalysis and constitute the active site: R68, R73, F90, and K92. Individual active site residues can significantly contribute to the activity of HigB. The basic nature of the positively charged residues Arg68 and Arg73 is thought to contribute to the charge stabilization of the negatively charged transition state [27]. The HigBs may also have specificity depending on the type of residue containing the phenyl ring. F90 might be important for the catalytic efficiency of HigB and facilitate the positioning of nucleotide substrates [28]. Taken together, the evidence shows that arginine and phenylalanine, which constitute the active site, will participate in the RNA cleavage reaction by organizing the active site and substrate geometry through an acid‐base mechanism. Lysine may only be related to additional charge stabilization in the catalysis without greatly affecting the activity of *S. pneumoniae* HigB in this study [31].

### HigBA peptide derivatives are promising new antimicrobial agents

The active site of HigB is not occluded, although the formation of the HigBA complex can block the toxicity of HigB. We focused on the neutralization of HigB toxin via binding with HigA and designed a peptide that mimics the binding site between HigB and HigA to estimate the potential of an antibiotic peptide through free toxin release by competition with HigB or HigA at the binding site. In our mutational study, the single point mutation of any interacting residue in the tight binding region of the HigBA complex did not result in HigB toxicity. However, the HigBA complex was fully disrupted by mutation of the binding region‐mimicking peptides containing numerous interacting residues; thus, a large increase in RFU was observed in the *in vitro* test. The active site of HigB is not blocked by HigA in the HigBA complex structure. And the active site and HigA binding region of HigB are distant from each other. Therefore, it can be inferred that the peptide blocks the toxicity of HigB allosterically. These notable characteristics are also observed in other HigBA complex structures. In *P. vulgaris* HigBA complex, *P. vulgaris* HigA does not cover the *P. vulgaris* HigB active site and only forms a minimal boundary junction [16]. Similarly, *V. cholerae* HigA does not interact with the active site of *V. cholerae* HigB but sufficiently neutralizes the *V. cholerae* HigB [15]. In general, HigB inhibition likely is not solely mediated by active site obstruction by HigA.

In our *in vivo* experiments, the peptides designed based on structural information about HigBA also successfully penetrated the cell membrane of *S. pneumoniae* and proved its growth arrest effect on *S. pneumoniae*. Therefore, we suggest the possibility of new antimicrobial agents based on structural and functional studies of the HigBA TA system, which has a completely new mechanism that does not exist in current antibiotics. HigB toxin does not exhibit toxicity when it is part of a TA complex because the HigA antitoxin completely blocks the active site of the HigB. However, when a peptide binds to a TA complex and interacts with its binding partners, the complex is disrupted by the peptide inhibitor and the toxin is released from the complex. Additionally, in the case of the VapBC and HicBA systems, the activity of the toxin is regenerated when an active site of the free toxin is exposed by the addition of a peptide mimicking the binding site [32, 33]. Taken together, our study establishes that the structurally designed peptides can function as bactericidal agents in *S. pneumoniae*. Furthermore, improvements in activity and stability obtained by modifying current candidate peptides using hydrocarbon α‐helix stapling or hydrogen bond surrogates will enable these antibiotic peptides as a new type of antimicrobial agent that targets specific pathogenic bacteria [14, 34, 35].

## Materials and Methods

### Cloning, mutation, expression, and purification

The genes encoding *S. pneumoniae* (strain TIGR4) HigB (*sp_1143*) and HigA (*sp_1144*) were amplified by polymerase chain reaction (PCR) using the primers HigB‐F, HigB‐R, HigA‐F, and HigA‐R (Table S1). The restriction enzymes *Nde1* and *Xho1* were used to cut the PCR products and vectors [pET‐28b(+) for HigB and pET‐21a(+) for HigA] (Novagen, Madison, WI, USA). The cloned plasmids of HigB and HigA were cotransformed into *E. coli* Rosetta (DE3) pLysS for the overexpression of the HigBA complex. The cells were grown in Luria‐Bertani (LB) medium containing 50 µg·mL^−1^ ampicillin and 30 µg·mL^−1^ kanamycin at 310 K until an OD_600_ of 0.5 was reached. The overexpression of the target proteins was induced by 0.5 mm isopropyl‐β‐D‐thiogalactopyranoside (IPTG) with an additional incubation for 4 h. The cells were harvested at 11,355 x g. The harvested cells were lysed by ultrasonication in buffer A (20 mm Tris/HCl, pH 7.9, 500 mm NaCl) containing 10% (v/v) glycerol. The entire lysate was centrifuged at 28 306 g for 1 h at 277 K. The supernatant was loaded onto an open Ni^2+^‐affinity column (Qiagen, Hilden, Germany), pre‐equilibrated with buffer A, and washed with buffer A containing 50 mm imidazole. The protein bound to the column was eluted by an imidazole gradient (50–700 mm). For further purification using ion‐exchange chromatography, the buffer containing the target proteins was changed to buffer B (50 mm MES, pH 6.0). Proteins were loaded onto a HiTrap SP column (GE Healthcare) pre‐equilibrated with buffer B and eluted with a NaCl gradient (0–1.5 m). As a final step, size‐exclusion chromatography was conducted using a HiLoad 16/600 Superdex 200 prep‐grade column (GE Healthcare) in a final buffer (50 mm MES, pH 6.0, 500 mm NaCl). The purified proteins were confirmed by sodium dodecyl sulfate–polyacrylamide gel electrophoresis and concentrated using an Amicon Ultra centrifugal filter unit (Merck Millipore, Burlington, MA, USA) in preparation for crystallization. The HigBA complex displaced by selenomethionine (SeMet) (Calbiochem, San Diego, CA, USA) was expressed and purified by the same procedure, except M9 medium containing extra essential amino acids was used for cell growth.

For the ribonuclease activity assay of HigB, 8 M urea was added to purified HigBA complex as a denaturant. The final buffer containing 8 m urea and HigBA complex was incubated at 293 K for 4 h. Size‐exclusion chromatography was conducted on the denatured HigBA complex using a HiLoad 16/600 Superdex 200 prep‐grade column (GE Healthcare). The eluted fraction containing HigB was diluted to remove the denaturant and enable refolding, and the denatured HigB was diluted by a factor of one thousand with the final buffer. Mutated forms of HigB were constructed and subjected to a ribonuclease activity assay. Ten residues of HigB were mutated to alanine (F17A, M20A, D40A, L44A, D61A, E66A, R68A, R73A, F90A, and K92A) (Table S1) using an EZ Change Site‐Directed Mutagenesis Kit (Enzynomics, Daejeon, South Korea). The protein complexes of HigB mutants with the WT HigA were expressed and purified by the same procedure used for the WT HigBA. Each HigB mutant was generated by denaturation and refolding by the same procedure used for the WT HigB.

A HigA construct consisting of residues 19 to 97 was used for the NMR experiments. The genes encoding HigA^19–97^ were amplified by PCR using primers HigA‐F ^19–97^ and HigA‐R (Table S1) and cloned into pET‐28b(+) (Novagen), resulting in a twenty‐residue tag (MGSSHHHHHHSSGLVPRGSH) at the N terminus. The plasmid encoding HigA^19–97^ was transformed into *E. coli* Rosetta (DE3) pLysS for overexpression, and the cells were grown in M9 minimal medium supplemented with 1 g·L^−1^ [^15^N] ammonium chloride and 1 g·L^−1^ [^13^C] glucose as the sole nitrogen and carbon sources, respectively. The purification procedure was the same as that used for the complex. To remove the N‐terminal tag, thrombin was added to the purified protein (10 units·mL^−1^) and incubated at 293 K overnight. Finally, size‐exclusion chromatography was conducted using a HiLoad 16/600 Superdex 200 prep‐grade column (GE Healthcare) in buffer (50 mm MES, pH 6.0, 100 mm NaCl). The concentration of HigA^19–97^ was measured based on the Bradford method [36] because of the absence of aromatic amino acid residues.

### Crystallization, X‐ray data collection, and structure determination

Crystals of the HigBA complex were grown using the sitting‐drop vapor diffusion method at 293 K by mixing equal volumes (0.5 μL each) of the protein solution (11 mg·mL^−1^ in 50 mm MES, pH 6.0, 500 mm NaCl) and the reservoir solution (20% (w/v) PEG 1000, 0.1 m potassium phosphate monobasic/sodium phosphate dibasic, pH 6.2, and 0.2 m sodium chloride). A cryoprotectant solution consisting of 25% (v/v) glycerol was added to the reservoir solution for data collection under a liquid nitrogen stream. The crystal was vitrified in the cold nitrogen gas stream, and the data were collected using an ADSC Quantum Q270r CCD detector at beamline 5C of the Pohang Light Source, Republic of Korea. Crystals of HigBA belonged to the *monoclinic* space group P2_1_, with unit cell parameters of *a *= 74.903 Å, *b *= 74.403 Å, *c *= 98.321 Å, α = γ = 90.00°, β = 90.08° for the native complex and *a *= 74.575 Å, *b *= 67.038 Å, *c = *87.717 Å, α = γ = 90.00°, β = 94.22° for the SeMet‐labeled complex. The structure of the HigBA complex was first phased at 2.80 Å resolution by single‐wavelength anomalous dispersion using SeMet. The structure of the HigBA complex was calculated using 2.30 Å resolution diffraction data of the native complex with a molecular replacement based on the SeMet protein model. The detailed statistical information is described in Table S2. *PHENIX* [37] was first used to automatically build a residue, and the remaining residues were built in *Coot* [38] to provide the final model. The *R*_work_ and *R*_free_ [39] values were calculated by *REFMAC* and *PHENIX* for refinement [37, 40]. The overall geometry was validated using molprobity [41]. Figures were generated using pymol [42]. The electrostatic potential surfaces were calculated using the Adaptive Poisson‐Boltzmann solver (APBS) method [43].

### Electrophoretic mobility shift assay

The binding affinities of HigA and HigBA to the palindromic sequence in their own operator region were estimated by EMSA. Typically, type II antitoxins interact with DNA via symmetric palindromic stretches that are approximately 15 − 30 nucleotides long called inverted repeats. For *higBA*, we were able to locate several inverted repeats within the promoter region. Four DNA duplexes were selected and termed Pal‐I, Pal‐II, Pal‐III, and Pal‐IV (Table S3). Control experiments were also performed with DNA ‘X’ and the two other palindromic sequences ‘A’ and ‘B’ (Table S3). Purified DNA duplexes were purchased from Bioneer (Daejeon, South Korea). Reaction mixtures (10 µL) containing 0.01 mm of duplex DNA and different protein concentrations (a series of protein concentrations, with the HigA dimer and HigBA heterotetramer ranging from 0 to 0.6 mm) were prepared in binding buffer (50 mm MES, 500 mm NaCl, pH 6.0) and incubated for 30 min at 293 K. The reaction mixtures were electrophoresed on a 0.5% agarose gel with 0.5x TBE for 20 min. The results were visualized using a Printgraph 2M (ATTO, Tokyo, Japan).

### Isothermal titration calorimetry measurements

Isothermal titration calorimetry (ITC) experiments were conducted using an iTC200 calorimeter (Malvern Instruments, Malvern, Worcestershire, UK) at 25 °C. The proteins and promoter dsDNAs were prepared in a buffer consisting of 50 mm MES, pH 6.0, and 100 mm NaCl for both HigA and HigBA. The DNA duplexes used in the ITC measurements were the same as those used in the EMSA (Table S3). Affinity experiments were conducted with the protein solution (10 μm; HigA dimer and HigBA heterotetramer, 320 μL) in the cell and the dsDNA solution (600 μm) as the injected titrant. In total, 19 injections performed at 180‐s intervals were used for data collection. The MicroCal Origin software was used for curve fitting to calculate the binding affinity (*K_d_*), the enthalpy of binding (*△H*), the entropy of binding (△*S*), and the stoichiometry (*n*). The raw data were fitted using a one‐site binding model. The Gibbs‐free energies (△*G*) were calculated using the standard equation △*G =* △*H* − *T*△*S*.

### NMR spectroscopy

For the NMR analysis, HigA^19–97^ was uniformly labeled with ^13^C and ^15^N and prepared in 20 mm MES, pH 6.0, 50 mm NaCl, 1 mm EDTA, 0.1 mm PMSF, and 10% D_2_O. All NMR measurements were conducted at 308 K using an AVANCE 800 MHz spectrometer equipped with a cryogenic probe (Bruker BioSpin, Billerica, MA, USA). ^1^H, ^13^C, and ^15^N chemical shift assignments for the backbone nuclei were obtained using 2D ^1^H‐^15^N HSQC and a set of triple resonance experiments to obtain the HNCO, HN(CA)CO, HNCA, HN(CO)CA, HNCACB, and HN(CO)CACB spectra. Data were processed using NMRPipe and nmrDraw [44] and further analyzed using NMRviewJ [45].

### NMR titration experiment

The NMR titration experiments on ^15^N‐labeled HigA^19–97^ with the promoter dsDNA Pal‐III (Table S3) were conducted by acquiring the ^1^H‐^15^N HSQC spectra. Throughout the NMR titration experiments, the concentration of the HigA^19–97^ dimer was maintained at 0.5 mm, and the concentration of the promoter dsDNA was increased from 0 to 0.1 mm. The chemical shift perturbation (CSP) was calculated and analyzed using Equation 1, where Δδ_N_ and Δδ_H_ are the CSP values of the amide nitrogen and proton, respectively. The intensity ratio was calculated by dividing the peak intensity of the last spectrum by the peak intensity of the first spectrum:

$$\Delta \delta_{\text{avg}}=\;{\left[\left(0.2\times △\delta_{N}^{2}+△\delta_{H}^{2}\right)/2\right]}^{1/2}.$$

### *In silico* HigBA‐DNA docking

*In silico* molecular docking to examine the interaction between Pal‐III and HigBA was performed using the high ambiguity‐driven protein–protein DOCKing algorithm (HADDOCK) [46]. The 20‐base pair promoter DNA was modeled using a 3D‐DART server [47]. Residues S57, T69, and Q72 were defined as ‘active residues’ based on the NMR titration results. Residues A2, C12, T13, and T14 of Pal‐III‐F, T7, T8, A9, and C19 of Pal‐III‐R, Q47, R63, and T75 of HigA were additionally designated to generate a rational interface for HTH recognition contact between the protein and DNA. Passive residues were defined automatically as the residues surrounding the active residues.

### *In vitro* ribonuclease assay

The ribonuclease activity of HigB and HigB mutants was verified using an RNase Alert Kit (IDT, Coralville, IA, USA) as previously reported (32).

### Kill‐and‐rescue assay

Genes encoding HigB mutants (R68A, R73A, F90A, and K92A) were amplified and cloned into pBAD33. The compatible plasmid pairs (pBAD33 and pET‐21a(+)) with and without each HigB mutant and HigA were cotransformed into BL21 (DE3) competent cells and grown overnight at 37 °C. The overnight cultures were diluted by 100‐fold in 20 ml of fresh M9 minimal medium containing 25 µg·mL^−1^ chloramphenicol and 50 µg·mL^−1^ ampicillin. The bacteria were then streaked onto LB medium plates supplemented with inducer l‐arabinose (0.2% w/v) or 0.5 mm IPTG. The plates were incubated overnight at 37 °C before examination of the cell growth.

### Circular Dichroism spectroscopy

Circular Dichroism (CD) measurements were conducted to verify the structural integrity of the HigB and HigB mutants using a Chirascan plus spectropolarimeter (Applied Photophysics, Leatherhead, UK) at 20°C in a cell with a 1‐mm light path. CD scans were performed from 260 nm to 190 nm with a 1 nm bandwidth and a scan speed of 100 nm·min^−1^. Three scans were averaged, followed by subtraction of the solvent signal.

### Peptide design and addition to the HigBA complex

Three peptides that mimicked the binding interface region between HigB and HigA were designed and purchased from AnyGen (GwangJu, South Korea, www.anygen.com). Theoretically, mimicked peptides compete with the original protein for binding, and if peptides occupy the binding site, the toxin is freely released to digest RNA, which results in the detection of fluorescence in the fluorescence quenching assay. To disrupt the binding interface between HigB and HigA and restore the toxicity of HigB, peptides were added to the HigBA complex (Table S4).

### Antimicrobial activity test

The antimicrobial activity of the mimicking peptides was evaluated as previously reported (32). The activity of the peptides against Gram‐positive *S. pneumoniae* TIGR4 (ATCC^®^ BAA‐334^TM^) was tested. Several scrambled peptides with the same amino acid sequence as the HigB α2 helix‐mimicking peptide were also tested (Table S4).

### *In vivo* cell growth assay

For the cell growth assay, *S. pneumoniae* TIGR4 was grown for 10 h without agitation at 310 K in Todd Hewitt Broth (THB) in air containing 5% CO_2_ until the mid‐exponential phase (OD_600_ of 0.5). The 10 h of cultures were inoculated into fresh medium for assays. The cells were incubated at 37 °C for 10 h, and growth was monitored at 1 h of intervals. The average OD_600_ values, along with the standard error of the mean, were plotted in MicroCal Origin software.

### Generation of *S. pneumoniae* D39 containing mutated *HigBA*

For the electroporation experiments, *S. pneumoniae* D39 cells were grown at 37 °C in THB until the early‐exponential phase (OD_600_ of 0.1–0.2). They were harvested and washed twice using electroporation medium containing 0.5 M sucrose, 7 mm potassium phosphate, pH 7.5, and 1 mm MgCl_2_. Subsequently, they were concentrated ten times in the same medium mentioned above. Then, 0.1 mL of the concentrated cell suspension was poured into a Bio‐Rad cuvette along with 1 μg·mL^−1^ plasmid DNA. The mixture was incubated on ice for 10 min. A single impulse of current was supplied with the gene pulse apparatus set at 6.25 kV and 25 μF. The electro‐transformed cells were then incubated at 37 °C with constant shaking for approximately 1 h. Finally, the desired cells were obtained using selective media.

### Flow cytometry

*Streptococcus pneumoniae* TIGR4 cells were cultured in a 96‐well flat‐bottom plate containing THB (MBcell) with various concentrations of peptide using a serial dilution method. At two time points (1 h later and 10 h later), bacteria cells were harvested and stained with a LIVE/DEAD BacLight Kit (Thermo Fisher Scientific, Waltham, MA, USA) containing SYTO^®^ 9 and PI. A positive control was prepared by measuring fresh cells, and a negative control was prepared by incubating cells with 70% isopropyl alcohol for 15 min before washing. For staining, the cells were incubated with 5 μm SYTO^®^ 9 and 30 μm PI at room temperature and protected from light for 15 min. The cells were analyzed with an LSRFortessa cell analyzer (BD Bioscience, San Jose, CA, USA), and the data were analyzed using FlowJo software (TreeStar, Ashland, OR, USA). *S. pneumoniae* D39 cells and four active site mutants containing *S. pneumoniae* D39 cells were subjected to the same treatment used for the *S. pneumoniae* TIGR4 cells after 10 h of incubation with peptides.

### Confocal images

*S. pneumoniae* cells were cultured in THB during exponential growth (OD_600_ between 0.4 and 0.6). The cells were diluted with Difco^TM^ Mueller Hinton Broth (BD Bioscience) to obtain a 10% cell suspension. The cell suspension was incubated with 1 μm peptide samples conjugated with fluorescein for 30 min, and then, to remove the peptides bound to the cell surface, 1 mg·mL^−1^ trypsin from bovine pancreas (Sigma Life Sciences) was added for quenching. After 30 min, the cells were washed with PBS and then analyzed with a Leica TCS SP8 (Leica Microsystems, Wetzlar, Germany) confocal microscope.

## Conflict of Interest

The authors declare no conflict of interest to declare.

## Author contributions

SMK, CJ, and DHK conceived the study. SMK, CJ, and DHK designed the experiments. SMK, CJ, DHK, and SWH performed the experiments. SMK, CJ, and DHK analyzed the data. SMK, CJ, DHK, and SWH wrote the manuscript. SMK, DHK, CJ, SJP, and BJL edited the manuscript.

## Supporting information


**Fig. S1.** Ribbon representation and 90°‐rotated diagrams of (A) HigBA heterotetramer, (B) HigBA dimer, (C) HigB and (D) HigA. PyMOL was used to generate Fig. S1.
**Fig. S2.** Molecular weight estimates of HigBA and HigA19 − 97 obtained by size‐exclusion chromatography.
**Fig. S3.** Full‐length sequence comparison and percentages of amino acid identities of (A) HigBs and (B) HigAs.
**Fig. S4.** EMSA study using other palindromic sequences ‘A’ and ‘B’ and control DNA ‘X’.
**Fig. S5.** ITC results with palindromes in the *higBA* promoter region.
**Fig. S6.** 2D 1H‐15N HSQC spectra of full‐length HigA and HigA19‐97.
**Fig. S7.** Validation tests for toxin mutants and peptide mimetics.
**Fig. S8.** Active site of HigB from *S. pneumoniae*.
**Table. S1.** Primers used for cloning.
**Table. S2.** Data collection and refinement statistics for SeMet‐substituted and native structures.
**Table. S3.** DNA used in EMSA, ITC and NMR titration.
**Table. S4.** Peptides used to disrupt the binding interface of HigBA.
