# Supplementary material for: Structure‐based design of peptides that trigger Streptococcus pneumoniae cell death
Source: FEBS J. 2020 Aug 31;288(5):1546–64. doi: 10.1111/febs.15514 (PMC7984235; doi:10.1111/febs.15514)
Supplement: Supplementary file 1 — Fig. S1. Ribbon representation and 90°‐rotated diagrams of (A) HigBA heterotetramer, (B) HigBA dimer, (C) HigB and (D) HigA. PyMOL was used to generate Fig. S1. Fig. S2. Molecular weight estimates of HigBA and HigA19 − 97 obtained by size‐exclusion chromatography. Fig. S3. Full‐length sequence comparison and percentages of amino acid identities of (A) HigBs and (B) HigAs. Fig. S4. EMSA study using other palindromic sequences ‘A’ and ‘B’ and control DNA ‘X’. Fig. S5. ITC results with palindromes in the higBA promoter region. Fig. S6. 2D 1H‐15N HSQC spectra of full‐length HigA and HigA19‐97. Fig. S7. Validation tests for toxin mutants and peptide mimetics. Fig. S8. Active site of HigB from S. pneumoniae. Table. S1. Primers used for cloning. Table. S2. Data collection and refinement statistics for SeMet‐substituted and native structures. Table. S3. DNA used in EMSA, ITC and NMR titration. Table. S4. Peptides used to disrupt the binding interface of HigBA. [file FEBS-288-1546-s001.zip › febs15514-sup-0001-Supinfo.pdf]

FEBS PRESS
science publishing by scientists
WILEY
The FEBS Journal
www.febsjournal.org

# Structure-based design of peptides that trigger *Streptococcus pneumoniae* cell death

Sung-Min Kang, Chenglong Jin, Do-Hee Kim, Sung Jean Park, Sang-Woo Han and Bong-Jin Lee

DOI: 10.1111/febs.15514

# Figure. S1

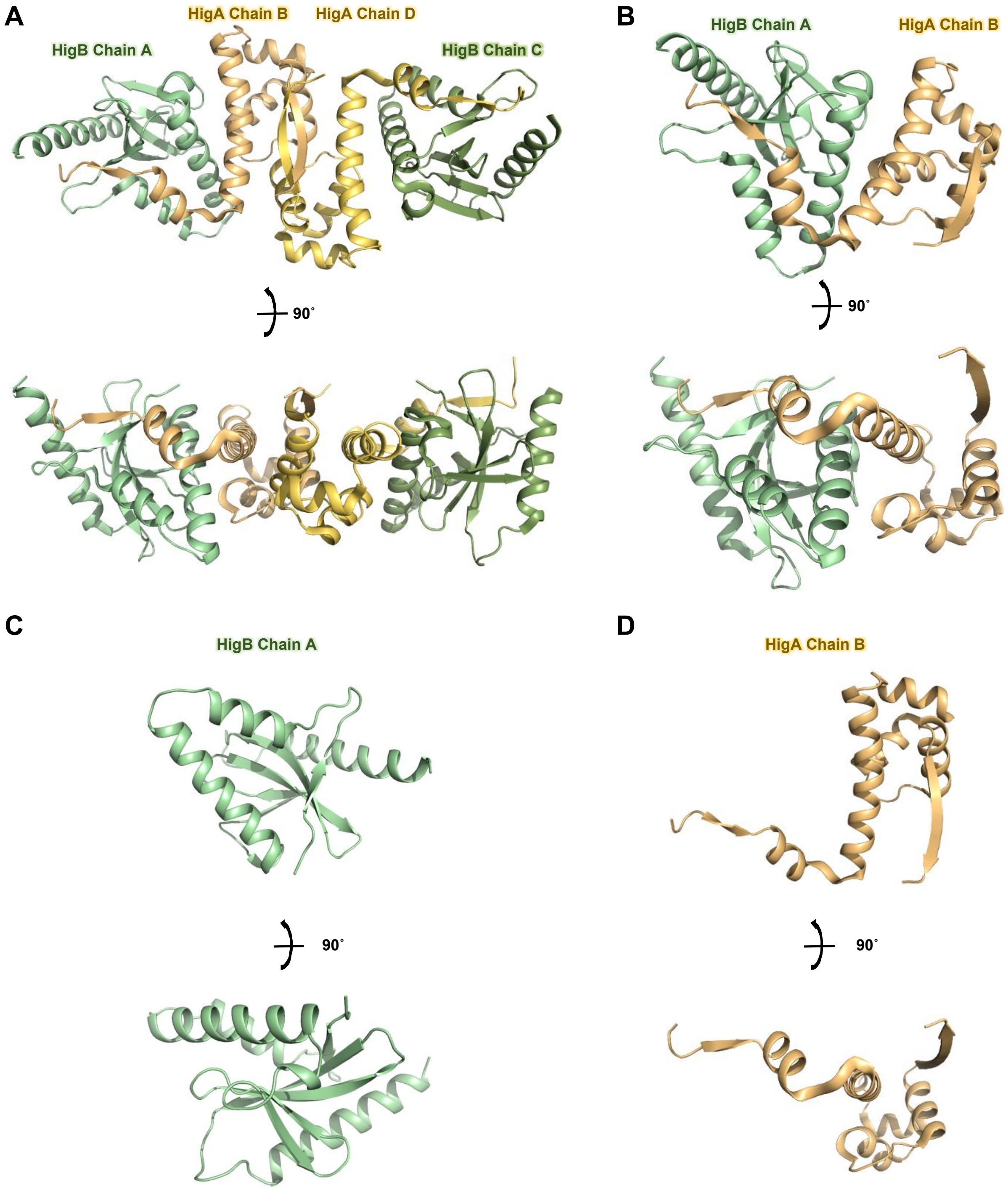


**Fig. S1.** Ribbon representation and 90°-rotated diagrams of **(A)** HigBA heterotetramer, **(B)** HigBA dimer, **(C)** HigB and **(D)** HigA. PyMOL was used to generate Fig. S1.

**Figure. S2**

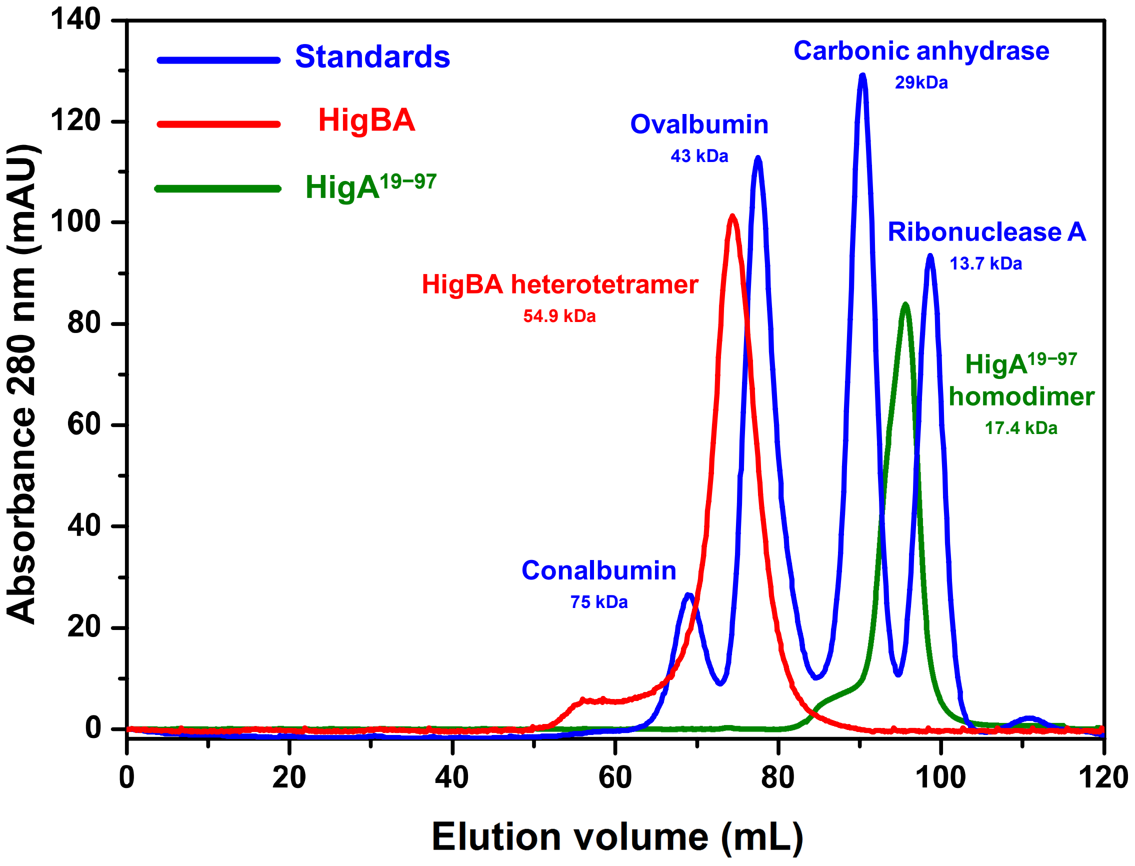


**Fig. S2.** Molecular weight estimates of HigBA and HigA$^{19–97}$ obtained by size-exclusion chromatography. Each sample was fractionated on a Superdex 200 pg size-exclusion column. Chromatograms were overlaid and are displayed with molecular weight calibration standards, which are indicated as follows: 75 kDa, 43 kDa, 29 kDa and 13.7 kDa. The overlay shows that the molecular weight of HigBA is between 43 kDa and 75 kDa. The molecular weight of heterotetrameric HigBA (54.9 kDa) is in this range. The overlay also shows that the molecular weight of HigA$^{19–97}$ is between 13.7 kDa and 29 kDa. The molecular weight of homodimeric HigA$^{19–97}$ (17.4 kDa) is in this range.

# Figure. S3

A

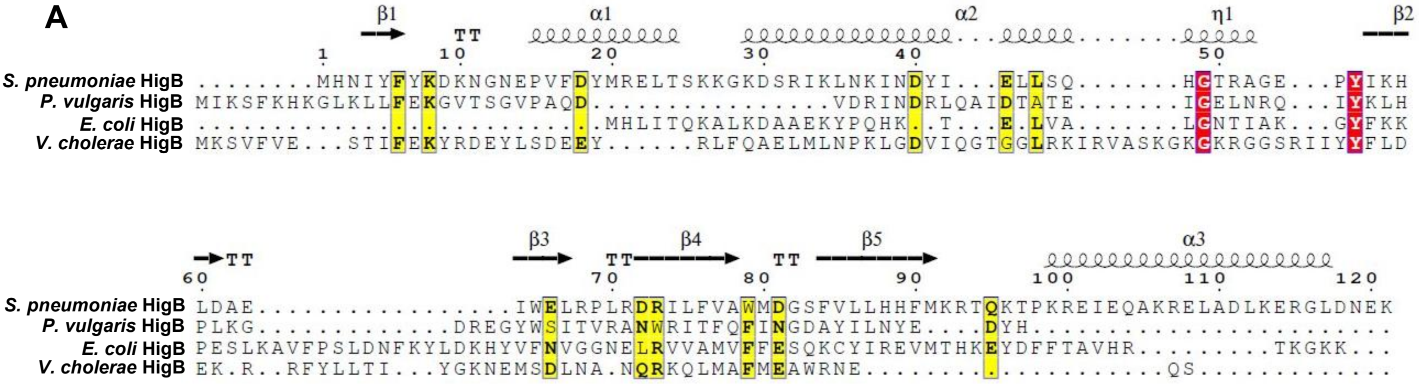


| Protein name | Sequence identity with *S. pneumoniae* HigB (%) |
|---|---|
| ***P. vulgaris*** **HigB** | 14 |
| ***E. coli*** **HigB** | 4 |
| ***V. cholerae*** **HigB** | 10 |

| Protein name | Sequence identity with *S. pneumoniae* HigA (%) |
|---|---|
| ***P. vulgaris*** **HigA** | 12 |
| ***E. coli*** **HigA** | 5 |
| ***V. cholerae*** **HigA** | - |

B

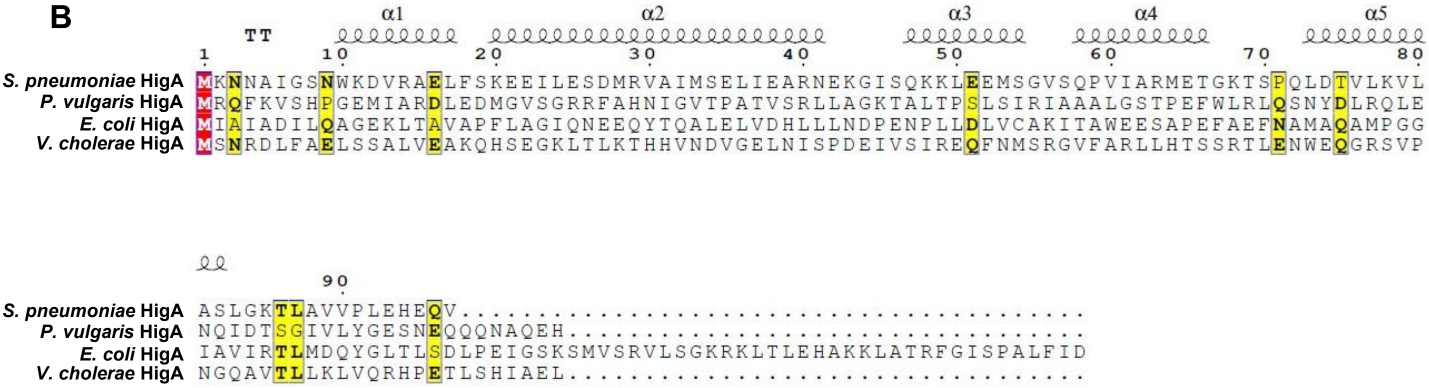


**Fig. S3.** Full-length sequence comparison and percentages of amino acid identities of **(A)** HigBs and **(B)** HigAs. Structural information of *S. pneumoniae* HigBA was used as the top secondary structures, and the description and sequence numbering on the topside correspond to *S. pneumoniae* HigBA. Residues showing similarity are highlighted in red and yellow. The three previously reported structures of HigBA from *Proteus vulgaris* (PDB code 4MCT) (Fig. 2A) [16], *Escherichia coli* (PDB code 5IFG) (Fig. 2B) [17] and *Vibrio cholera* (PDB code 5JAA) (Fig. 2C) [15] were compared with the newly obtained structure of HigBA from *S. pneumoniae* (PDB code 6AF4) (Fig. 2D). Alignments of amino acid residues were carried out using ClustalW [20] and visualized using ESPript 3.0 [21].

## Figure. S4

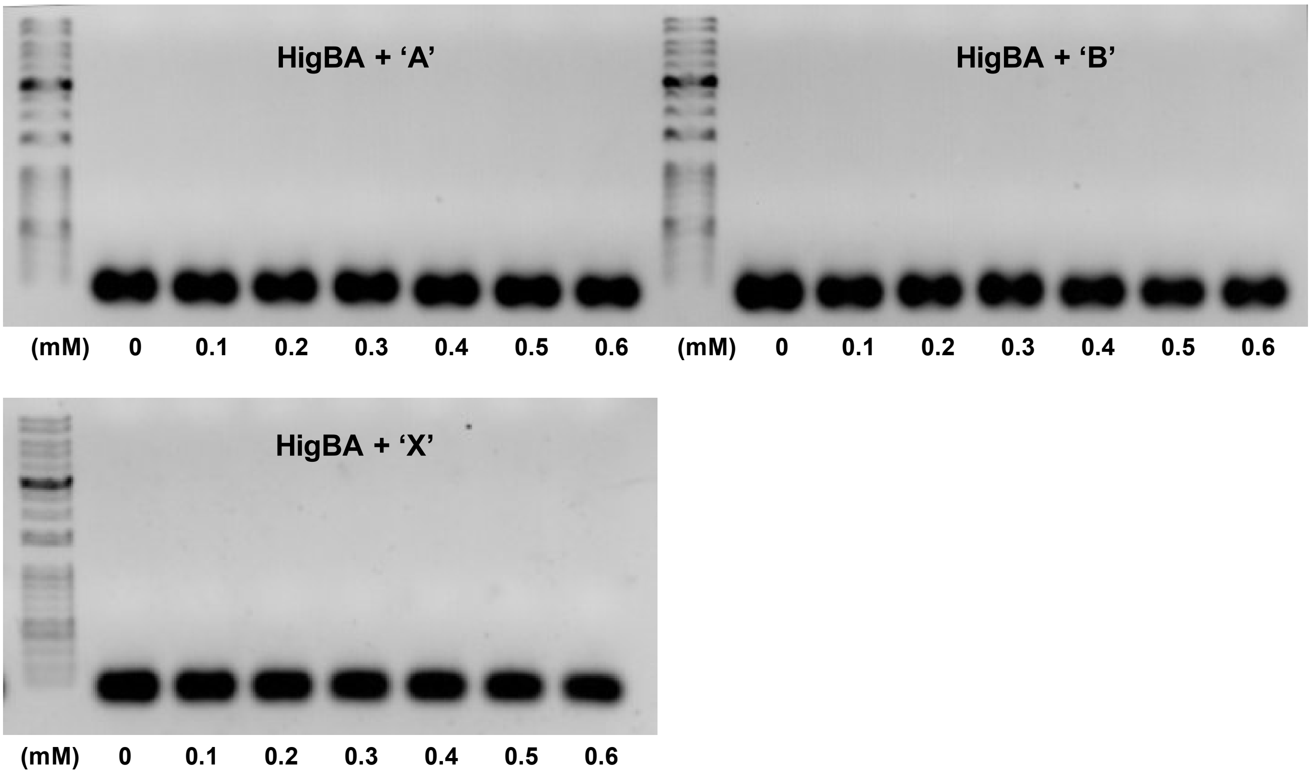


**Fig. S4.** EMSA study using other palindromic sequences ‘A’ and ‘B’ and control DNA ‘X’. Upper left: EMSA experiment testing the binding of HigBA to ‘A’. Upper right: EMSA experiment testing the binding of HigBA to ‘B’. Lower: EMSA experiment testing the binding of HigBA to DNA ‘X’. Each DNA concentration was 0.01 mM, and protein concentrations are indicated in each lane. Shown data are representative of three independent experiments.

**Figure. S5**

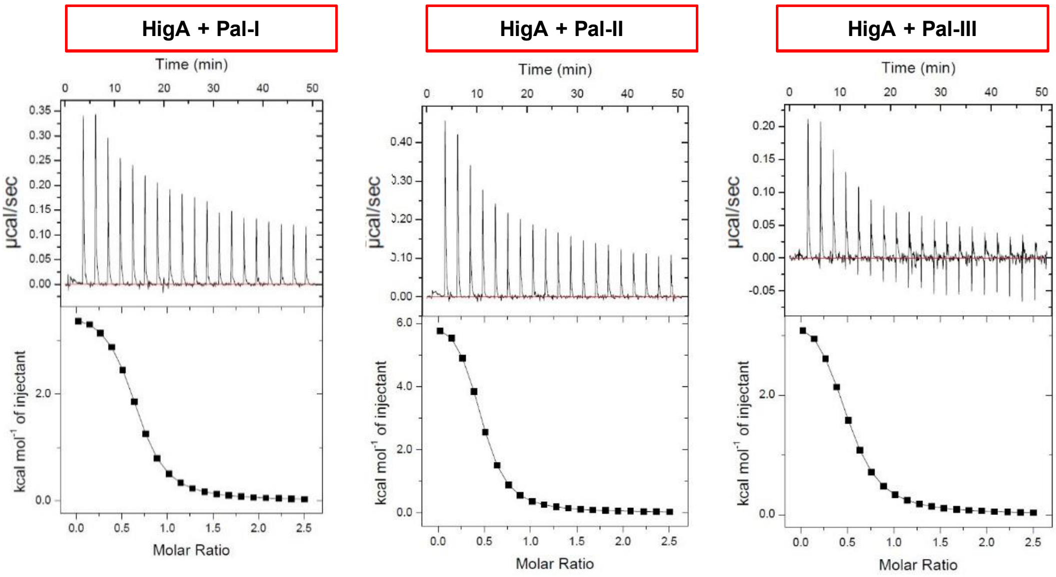


| DNA | *n (the binding stoichiometry)* | $K_d$ (μM) | △*H* (kcal mol-1) | *T*△*S* (kcal mol-1) | △*G* (kcal mol-1) |
|---|---|---|---|---|---|
| Pal-I | 0.53 ± 0.04 | 1.61 ± 0.02 | 6.4 ± 0.5 | 8.79 | -2.39 |
| Pal-II | 0.51 ± 0.02 | 2.53 ± 0.11 | 6.5 ± 0.3 | 7.53 | -1.03 |
| Pal-III | 0.50 ± 0.04 | 1.34 ± 0.01 | 5.7 ± 0.4 | 12.5 | -6.80 |

**Fig. S5.** ITC results with palindromes in the *higBA* promoter region. ITC assay of the binding of three palindromes (Pal-I, Pal-II and Pal-III) to HigA antitoxin. The binding parameters are described in the table (lower).

# Figure. S6

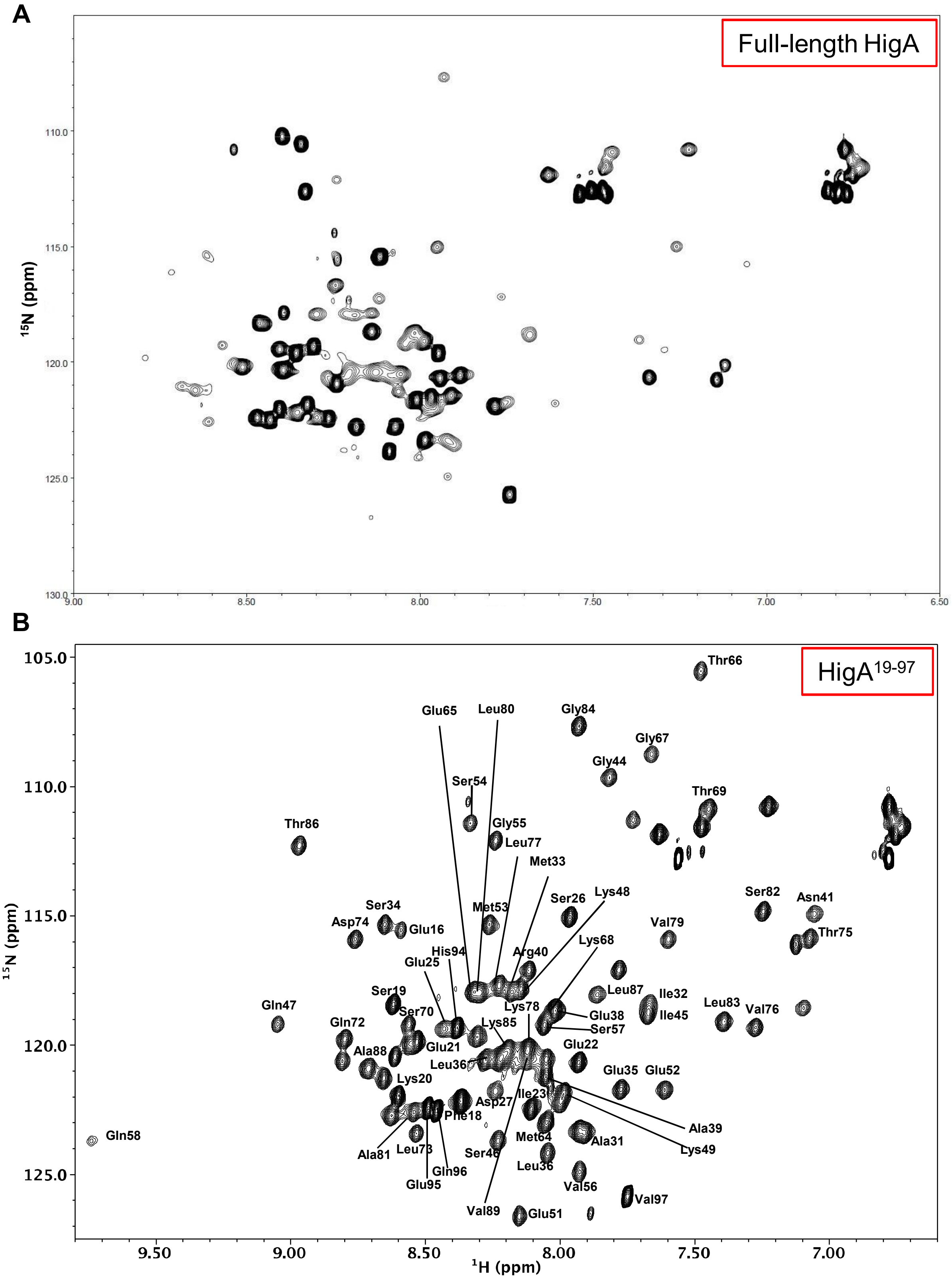


**Fig. S6.** 2D $^{1}H$-$^{15}N$ HSQC spectra of full-length HigA and HigA$^{19\text{-}97}$. **(A)** 2D $^{1}H$-$^{15}N$ HSQC spectrum of full-length HigA. The peaks of the full-length spectrum of HigA could not be assigned. **(B)** 2D $^{1}H$-$^{15}N$ HSQC spectrum of HigA$^{19\text{-}97}$. The assigned residues are indicated in the spectrum.

**Figure. S7**

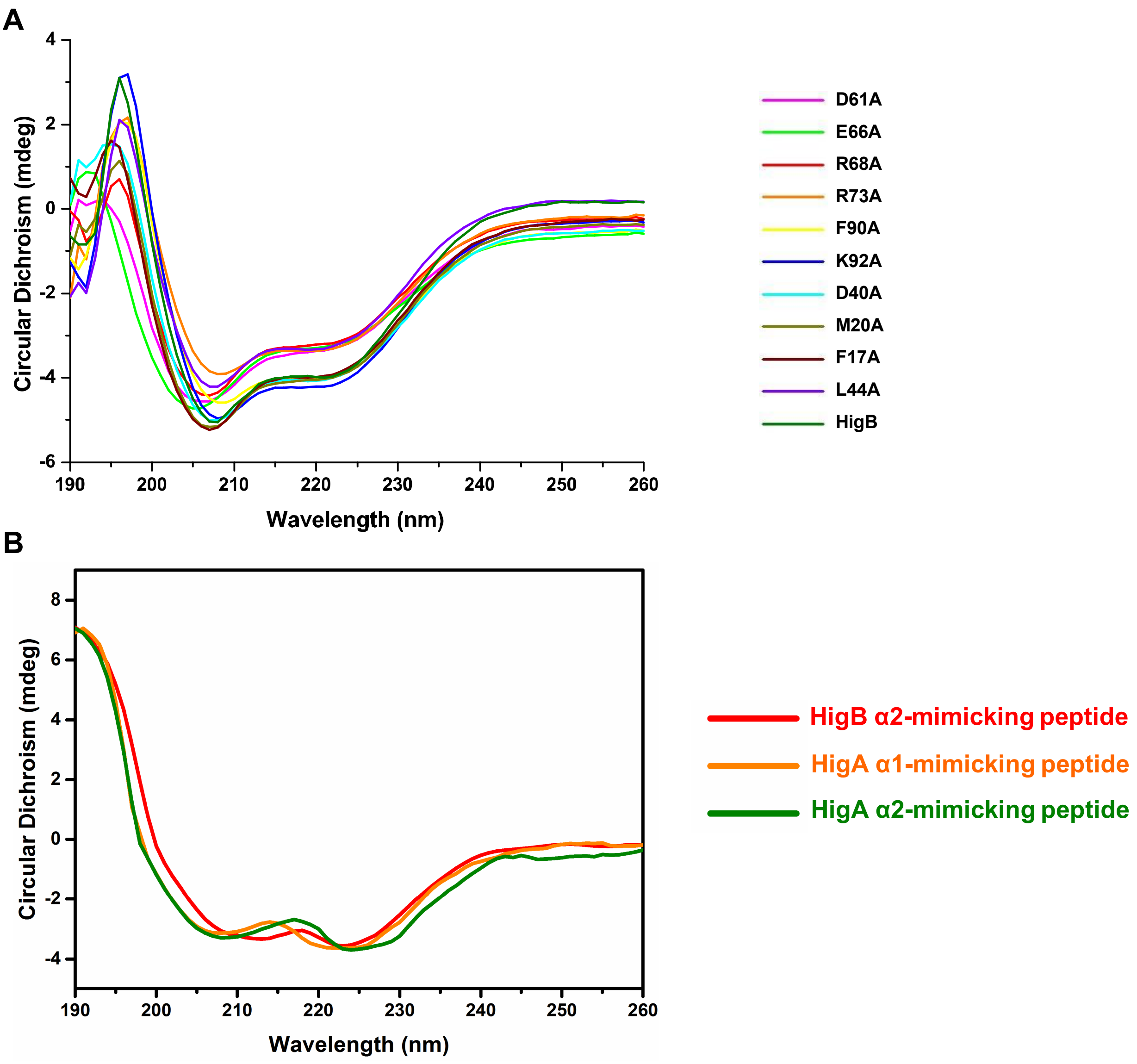


**Fig. S7.** Validation tests for toxin mutants and peptide mimetics. (**A** and **B**) Circular dichroism spectra of the **(A)** HigB mutants and **(B)** mimicking peptides in 50 mM MES, pH 6.0, 500 mM NaCl. All mutants regained their secondary structure after refolding.

**Figure. S8**

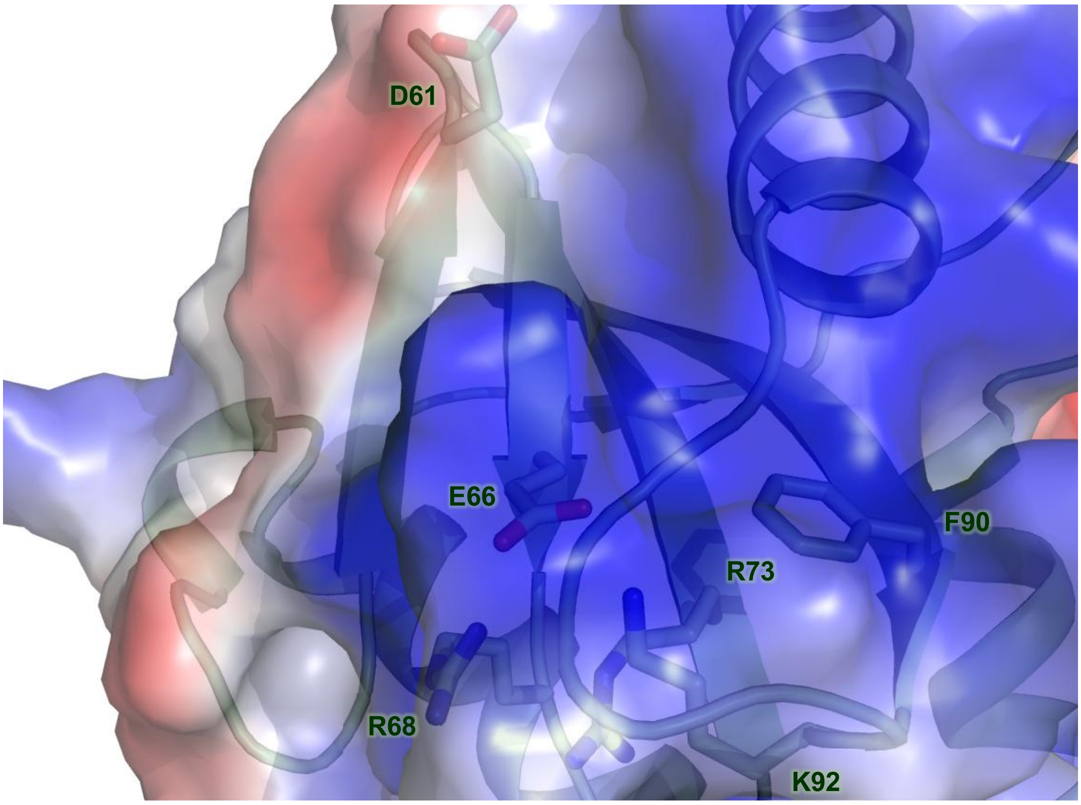


**Fig. S8. A**ctive site of HigB from *S. pneumoniae*. It is positively charged, indicating possible interactions with the negatively charged RNA phosphate backbone. PyMOL was used to generate Fig. S8.

**Table S1.** Primers used for cloning.

| Primer | Sequence |
|---|---|
| HigB-F[a] | 5'-GGAATTCCATATGATGCATAATATCTATTTTTA-3' |
| HigB-R[b] | 5'-CCGCTCGAGTTATTTTTCATTGTCTAAAC-3' |
| HigA-F[a] | 5'-GGAATTCCATATGATGAAAAATAATGCTATTGG-3' |
| HigA-R[b] | 5'-CCGCTCGAGTTAAACCTGCTCATGCTCTAATGGT-3' |
| F17A-F[a] | 5'-GGCAATGAGCCTGTTGCTGATTATATGCGAGAG-3' |
| F17A-R[b] | 5'-CTCTCGCATATAATCAGCAACAGGCTCATTGCC-3' |
| M20A-F[a] | 5'-CCTGTTTTTGATTATGCGCGAGAGCTTACCAGT-3' |
| M20A-R[b] | 5'-ACTGGTAAGCTCTCGCGCATAATCAAAAACAGG-3' |
| D40A-F[a] | 5'-CTTAATAAAATTAATGCTTATATTGAGTTGTTA-3' |
| D40A-R[b] | 5'-TAACAACTCAATATAAGCATTAATTTTATTAAG-3' |
| L44A-F[a] | 5'-AATGATTATATTGAGGCGTTAAGCCAACATGGA-3' |
| L44A-R[b] | 5'-TCCATGTTGGCTTAACGCCTCAATATAATCATT-3' |
| D61A-F[a] | 5'-TATATTAAGCATTTAGCTGCTGAAATTTGGGAG-3' |
| D61A-R[b] | 5'-CTCCCAAATTTCAGCAGCTAAATGCTTAATATA-3' |
| E66A-F[a] | 5'-GATGCTGAAATTTGGGCGCTGAGACCACTTAGA-3' |
| E66A-R[b] | 5'-TCTAAGTGGTCTCAGCGCCCAAATTTCAGCATC-3' |
| R68A-F[a] | 5'-GAAATTTGGGAGCTGGCACCACTTAGAGATAGA-3' |
| R68A-R[b] | 5'-TCTATCTCTAAGTGGTGCCAGCTCCCAAATTTC-3' |
| R73A-F[a] | 5'-AGACCACTTAGAGATGCAATTTTATTTGTTGCT-3' |
| R73A-R[b] | 5'-AGCAACAAATAAAATTGCATCTCTAAGTGGTCT-3' |
| F90A-F[a] | 5'-GTTTTACTGCATCATGCTATGAAAAGGACACAG-3' |
| F90A-R[b] | 5'-CTGTGTCCTTTTCATAGCATGATGCAGTAAAAC-3' |
| K92A- F[a] | 5'-CTGCATCATTTTATGGCAAGGACACAGAAAACA-3' |
| K92A-R[b] | 5'-TGTTTTCTGTGTCCTTGCCATAAAATGATGCAG-3' |
| HigA$^{19-97}$-F[a] | 5'-GGAATTCCATATGAGCAAAGAGGAAATTTTGG-3' |

[a, b] F and R represent forward and reverse, respectively. Enzyme sites are underlined.

**Table S2.** Data collection and refinement statistics for SeMet-substituted and native structures.

(a) Data collection details. Values in parentheses are for the highest-resolution shell.

| Data set | SeMet | Native |
|---|---|---|
| X-ray source | 5C beamline of PLS, Korea | 5C beamline of PLS, Korea |
| X-ray wavelength (Å) | 0.9794 | 0.9794 |
| Space group | $P2_1$ | $P2_1$ |
| Unit cell parameters | | |
| a, b, c (Å) | 74.575, 67.038, 87.717 | 74.903, 73.403, 98.321 |
| α, β, γ (°) | 90.0, 94.223, 90.0 | 90.0, 90.077, 90.0 |
| Resolution range (Å) | 50.0-2.80 | 50.0-2.30 |
| Molecules per ASU | 2 HigBA heterotetramers | 2 HigBA heterotetramers |
| Observed reflections ($>1\sigma$) | 235871 | 163245 |
| Unique reflections | 21414 | 45671 |
| $\langle I/\sigma(I)\rangle$ | 24.8 (1.13)[e] | 23.7 (1.61)[e] |
| Completeness (%) | 99.9 (99.6)[e] | 97.0 (100.0)[e] |
| Multiplicity[a] | 11.0 (9.0)[e] | 3.6 (3.8)[e] |
| $R_{merge}$ (%)[b] | 13.2 (139.8)[e] | 10.3 (70.9)[e] |
| $CC_{1/2}$, CC | (0.651, 0.888)[e] | (0.823, 0.950)[e] |

(b) Refinement statistics

| Data set | SeMet | Native |
|---|---|---|
| $R_{work}$[c] (%) | | 21.6 |
| $R_{free}$[d] (%) | | 25.4 |
| No. of atoms / average $B$ factor (Å$^2$) | | 6810 / 60.0 |

| | |
|---|---|
| RMSD[f] from ideal geometry | |
| Bond distance (Å) | 0.006 |
| Bond angle (°) | 1.135 |
| Ramachandran statistics | |
| Most favored regions (%) | 95.60 |
| Additional allowed regions (%) | 4.40 |
| Residues in disallowed regions (%) | 0.00 |
| PDB accession code | 6AF4 |

[a] $N_{obs}/N_{unique}$

[b] $R_{merge} = \Sigma\ (I - \langle I \rangle)/\ \Sigma \langle I \rangle$

[c] $R_{work} = \Sigma_{hkl}\ ||F_{obs}| - k\ |F_{calc}||/\ \Sigma_{hkl}\ |F_{obs}|$

[d] $R_{free}$ was calculated in the same way as $R_{work}$ but with 5% of the reflections excluded from the refinement.

[e] Values in parentheses indicate the highest-resolution shell.

[f] Root mean square deviation (RMSD) was calculated with REFMAC.

**Table S3.** DNA used in EMSA, ITC and NMR titration.

| DNA | Sequence |
|---|---|
| Pal-I-F[a] | AGCATCTAGGAAACTAGGTGCT |
| Pal-I-R[b] | AGCACCTAGTTTCCTAGATGCT |
| Pal-II-F[a] | AACTTAAAAGTATTTACAAACAATAACTTTTAGGTT |
| Pal-II-R[b] | AACCTAAAAGTTATTGTTTGTAAATACTTTTAAGTT |
| Pal-III-F[a] | TATTTTAATAACTTAAAAGT |
| Pal-III-R[b] | ATAAAATTATTGAATTTTCA |
| Pal-IV-F[a] | TAGGTTATAATTGTTATTAGGAA |
| Pal-IV-R[b] | TTCCTAATAACAATTATAACCTA |
| ‘X’-F[a] | GATTTTTTTTGATTTTTTT |
| ‘X’-R[b] | AAAAAAATCAAAAAAAATC |
| ‘A’-F[a] | TATTTTAATAACTTAAAAGT |
| ‘A’-R[b] | ACTTTTAAGTTATTAAAATA |
| ‘B’-F[a] | TAATAGAATAATAAGTATCACTCCTTTA |
| ‘B’-R[b] | TAAAGGAGTGATACTTATTATTCTATTA |

[a, b] F and R represent forward and reverse, respectively.

**Table S4.** Peptides used to disrupt the binding interface of HigBA.

| Residues (start - end) | Mimicked protein | Mimicked region |
|---|---|---|
| SRIKLNKINDYIELL (31-45) | HigB | α2 |
| DINLLSENIKYIRKL (31-45 scrambled) | | |
| ENKIKLDRLISNYLI (31-45 scrambled) | | |
| IKDILYSNLRIKLEN (31-45 scrambled) | | |
| IYLLEIDINLKSKRN (31-45 scrambled) | | |
| KLKIISLEIDNYNRL (31-45 scrambled) | | |
| LNLISLKREDKYNII (31-45 scrambled) | | |
| LYNRIKIELSLNDIK (31-45 scrambled) | | |
| SINRDKKELYILLNI (31-45 scrambled) | | |
| YLRKNNSKELLIIDI (31-45 scrambled) | | |
| YSLINRIDLIKEKNL (31-45 scrambled) | | |
| SNWKDVRAELF (8-18) | HigA | α1 |
| EEILESDMRVAIMSELIE (21-38) | HigA | α2 |
